# Behavioral gender differences are reinforced during the COVID-19 crisis

**DOI:** 10.1038/s41598-021-97394-1

**Published:** 2021-09-28

**Authors:** Tobias Reisch, Georg Heiler, Jan Hurt, Peter Klimek, Allan Hanbury, Stefan Thurner

**Affiliations:** 1 Section for Science of Complex Systems, Center for Medical Statistics, Informatics and Intelligent Systems, Medical University of Vienna, 1090 Vienna, Austria; 2 Complexity Science Hub Vienna, 1080 Vienna, Austria; 3 Institute of Information Systems Engineering, TU Wien, 1040 Vienna, Austria; 4 Santa Fe Institute, Santa Fe, NM 85701 USA

**Keywords:** Computational science, Human behaviour

## Abstract

Behavioral gender differences have been found for a wide range of human activities including the way people communicate, move, provision themselves, or organize leisure activities. Using mobile phone data from 1.2 million devices in Austria (15% of the population) across the first phase of the COVID-19 crisis, we quantify gender-specific patterns of communication intensity, mobility, and circadian rhythms. We show the resilience of behavioral patterns with respect to the shock imposed by a strict nation-wide lock-down that Austria experienced in the beginning of the crisis with severe implications on public and private life. We find drastic differences in gender-specific responses during the different phases of the pandemic. After the lock-down gender differences in mobility and communication patterns increased massively, while circadian rhythms tended to synchronize. In particular, women had fewer but longer phone calls than men during the lock-down. Mobility declined massively for both genders, however, women tended to restrict their movement stronger than men. Women showed a stronger tendency to avoid shopping centers and more men frequented recreational areas. After the lock-down, males returned back to normal quicker than women; young age-cohorts return much quicker. Differences are driven by the young and adolescent population. An age stratification highlights the role of retirement on behavioral differences. We find that the length of a day of men and women is reduced by 1 h. We interpret and discuss these findings as signals for underlying social, biological and psychological gender differences when coping with crisis and taking risks.

## Introduction

Empirical research has long been concerned with assessing whether women and men behave differently in their daily lives. Behavioral differences were reported in communication behavior, visible for example in the different investment in biological offspring across women and men’s lifetimes^1^. Gender differences in mobility patterns do rise from a mix of cultural, infrastructure, resource, safety and socio-economic factors^2^. Psychological and cognitive and other non-reproductive differences have been studied for many decades, maybe even centuries, see e.g.^3^. Also differences in stress perception and respective coping mechanisms have been known to exist for a long time^4, 5^. Non-reproductive biological differences include women having shorter circadian rhythms^6^ and showing different co-morbidity patterns than men across their lifetimes^7^. Even in virtual societies of online game players strong behavioral gender differences were found. In particular, male and female players tend to behave differently in economic activities, their dealing with aggression and hostilities, and generally how they structure their social networks^8^.

In the last two decades it became possible to collect data on human behavior on a population-wide scale, see e.g.^10^. Some of that data has been used to investigate human responses to crisis and emergency situations^11–14^. Studying collective response to crisis is essential for catastrophy planning and coordination^15, 16^ and policy makers in health and safety^17^. Response to crisis also reveals human qualities that only surface when facing different kinds of actual or perceived danger^4, 14, 18, 19^.

Times of stress may alter social norms, socio-economic constraints, and “typical” behavior. It is *a priori* not clear if and how these changes increase or decrease behavioral gender differences. On the one hand, one might speculate that stress leads to a more universal behavior, where gender differences become less important and thus less pronounced^20^. On the other hand psychological gender differences might become amplified when coping with crisis^4, 21, 22^. A crisis such as the COVID-19 pandemic is an exceptional shock to social systems and can be seen as a *natural experiment* that allows us to investigate the impact of population-wide stress and its consequences on gender-specific changes in behavior. Such a natural experiment can be used to estimate the *resilience* of behavioral changes, i.e., how long it takes after the onset of a well-defined shock to return to pre-crisis patterns of behavior. This characteristic time might also be important for a better objective understanding of temporal changes of psychological effects after emergencies, which are usually studied with self-reported data at a few points in time^4, 5, 18, 21^.

At the end of 2019 the SARS-CoV2 virus emerged in China, causing an ongoing, world-wide pandemic. In response to sharply rising numbers during the “first wave”, on March 15^th^ the Austrian government introduced a severe nation-wide lock-down. The implemented non-pharmaceutical interventions (NPIs) included: school closures, restaurant closures, mandatory use of masks, incentives to use home-office, the complete prohibition of gatherings of any size, closure of all non-essential shops, and a general limitation of mobility. It was possible to leave the house for one of four reasons only: work that cannot be postponed, shopping for groceries, assisting others, and short recreational walks^23^. These measures led to a massive reduction of mobility as measured for example with cell-phone data^24^, or traffic counts^25^. The lock-down had severe consequences on public life: 58% of all Austrians who were in employment or self-employed reported that they were employed in a company that introduced home-office to at least some extent^26^, the number of people registered unemployed increased by 76%^27^, more than 1,300,000 persons were temporarily laid off^28^, and public life, such as theaters, cinemas, restaurants, bars, shopping-malls and even large parks, came to a halt.

The uncertainty of the situation, especially the threat of job-loss or additional childcare duties caused stress and anxiety in the Austrian population^29^. Right from the start, it led to the apprehension that women could be affected more by the lock-down due to additional childcare duties^30–32^, domestic violence^33^, employment in high exposure jobs, and simultaneously higher unemployment^32^. Austrian women were more affected by unemployment and partial layoffs^27^, surveys registered an increase of domestic violence^34^, and female scientists posted less pre-prints and started less projects^31^. It has been argued that during the COVID-19 pandemic “disproportionately affected women and widened gender inequalities across the globe”^35^. The fact that men and women react differently to stress and crises is not new. Women experience more stress^4, 36^ and employ relatively more active and problem-focused coping strategies^4, 5^, while men tend to emotion-focused coping, such as emotional avoidance^4^.

In this paper we want to understand the effects of the COVID-19 crisis on behavioral gender differences in five directions: Changes in communication patterns, changes in mobility, changes in food supply, changes in spending leisure time and changes in circadian rhythms as seen in digital traces. We discuss gender as more than the distinction between biologically different sexes, but as a socially constructed categorization^9^. The gender categories in our study are self-reported and are, for technical reasons, limited to female and male. We observe changes in the digital traces of humans in Austria that are shaped by the lived social experiences that are played out within specific contexts, constraints, and gendered opportunity structures. Many studies, including the present, empirically find behavioral and psychological gender differences. However, one should not interpret these findings as a manifestation of an inherent difference between men and women, but as a starting point to discuss the roots of different experiences of the pandemic that are lived by women and men. To this end we use longitudinal, nation-wide telecommunication data of 1.2 million cell-phones, covering about 15% of the entire Austrian population. The anonymized data covers the time period across the government interventions from February 1^st^ to June 29^th^. We split the observation time into 6 periods that characterize the different stages of the pandemic and the response to it. To control for differences between our sample composition and the demographics of Austria, and to relate behavioral changes to different phases of life, we stratify our results with respect to age. From this data we extract gender-specific features about communication patterns, such as the average interaction duration and the number of calls for all possible gender combinations of calling and being called. The data further allows us to characterize mobility. From location data we estimate the number of people shopping for food and the usage of recreational areas. Finally, we estimate circadian activity of telecommunication and internet usage, from which we estimate e.g. gender differences in sleeping patterns. Telecommunication data has been used earlier to study the effect of crisis and emergencies. They were used to detect crisis^37^, study communication patterns subsequent to different emergencies^11^, predict movement, e.g. subsequent to the Haiti earthquake 2010^12^, and to help explain the spread of SARS-CoV-2^16^.

**Figure 1:**
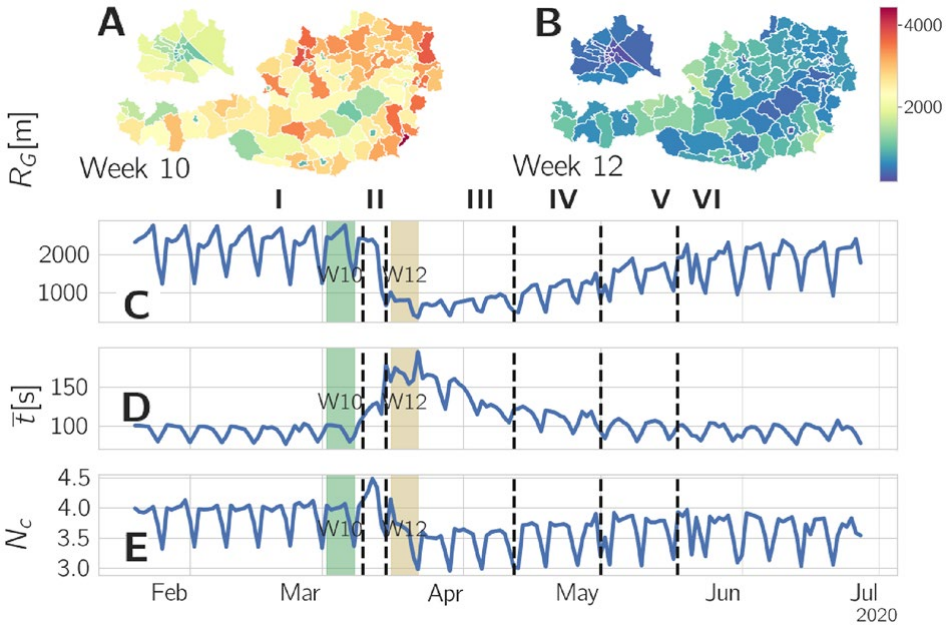
Population-wide response to the COVID-19 crisis. The maps show the mobility (radius of gyration, $R_G$) for calendar week (**A**) 10 and (**B**) 12 for Austria. The timeseries below outline the changes in (**C**) $R_G$, (**D**) the call duration per call $\bar{t}$, and (**E**) the number of calls per device $N_c$. During the lock-down mobility was drastically reduced throughout Austria. The call duration per call $\bar{t}$ increased dramatically and the number of calls, after a brief increase around the beginning of the lock-down, dropped below the pre-lock-down level.

Gender differences in human mobility and communication were studied in^1, 2^. In^1^ changes in communication behavior across age and gender were reported and in particular, how reproductive investments and preferred relationships of both sexes shift over a lifespan. It is a known fact that males tend to have their workplaces further away from home and thus generally move more, see e.g.^38^. Gender differences in mobility in Santiago de Chile are reported in^2^. There, significantly different movement behavior is found and is interpreted as a result of an interplay of socio-economic and urban factors. The gender specific behavioral response to seven terrorist attacks in six cities is investigated in^22^. They compare temporal mobile phone communication patterns in response to the attacks and report significant differences between the genders.

## Results

We partition the observation period from February 1st to June 29th 2020 into six periods: I Pre-awareness phase. The population is practically not yet aware of the presence of the disease in Austria. II Transition period from the announcement (March 12th) to the actual lock-down on March 16th. III lock-down until first easing of NPIs (April 13th). IV Period of some easing of NPIs. V Gatherings of more than 10 people are allowed, begins on May 1st. VI Back to normal, restaurants and businesses re-open. For more details, see SI Text S1. We analyze 454,000 women and 452,000 men, for a description of the data see Methods and SI Text S2.

### Overall behavioral changes during the lock-down

Figure 1 shows the effects of the lock-down. A reduction of mobility in the districts of Austria occurs from before the lock-down (panel A) to right after it (panel B). As a measure for mobility we use the median radius of gyration, $R_G$, see Methods and SI Text S3. $R_G$ captures the time weighted, spatial extent of an individuals trajectory. We observe a decrease of $R_G$ between 59% and 14%. Panel C shows the time evolution of $R_G$, averaged over all districts. After a sharp decline of almost 50% in phase III a rebound to almost pre-crisis levels is seen. In panel D we observe a more than 60% increase of call duration per call, $\bar{t}$. For a definition, see Methods. Panel E shows a brief increase of the number of calls per person, $N_{c}$, in the days just before the lock-down (phase II) followed by a 10% decrease. We now stratify these changes with respect to gender and age.

### Communication patterns

**Figure 2:**
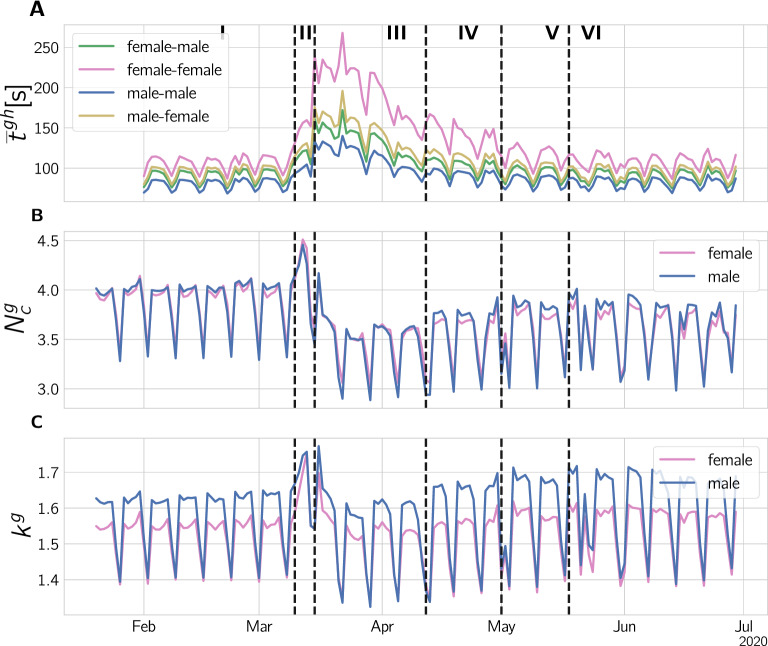
Gender-specific changes in communication behavior. (**A**) Median call duration of the four possible types of gender-specific calls, depending on who initiated the call and who received it. By mid-May pre-crisis levels are reached. Half-life times range from 17.3d in the female-female to 14.9 in the female-male case. (**B**) Number of calls originating from males (blue) and females (red). The median call duration peaks in phase III, particularly for female-female calls, whereas the number of calls assumes a minimum. Up to the end of the observation period, pre-crisis levels are not reached. (**C**) The number of communication partners, the degree $k^g(t)$, rises briefly and then drops below pre-crisis levels.

As proxies for the strength of social interactions, we first analyze the call duration per pair of interaction partners, $\bar{t}^{gh}(t)$, the number of calls, $N^{g}_c(t)$, and the number of calling partners per user, $k^g(t)$, see Methods. The superscripts indicate gender, *g* represents the gender of the caller; *h* is the gender of the called.

Figure 2 depicts the situation over time. In panel A we see a massive increase of calling times for the different gender combinations in phase II and the beginning of III. For the female-female calls we observe an increase of up to 140%, female-male and male-female rise by up to 81% and 97%, respectively, and male-male calls increase up to 66%. We find that calls involving women are generally longer than those involving men. Moreover, the call time increase is larger when women are involved.

Calling times decrease gradually and reach pre-crisis levels in phase VI. This decay can be fitted with an exponential function. The exponents of the fits translate into corresponding “half-life” times, which are $t_{1/2, mm} = 15.9$d for male-male and $t_{1/2, ff} = 17.3$d for female-female interactions, the mixed interactions have half-life times of $t_{1/2, mf} = 15.5$d and $t_{1/2, fm} = 14.5$d for male-female and female-male interactions, respectively. For details, see SI Text S4. Call times show a pronounced bias towards female initiated calls being longer. In phase I, female originated calls were 10% longer than male originated, and up to 30% longer on weekdays in phase III. From its maximum in phase III, the gender ratio continuously declines to normal levels in phase V, see Supplementary Fig. 14 A.

**Figure 3:**
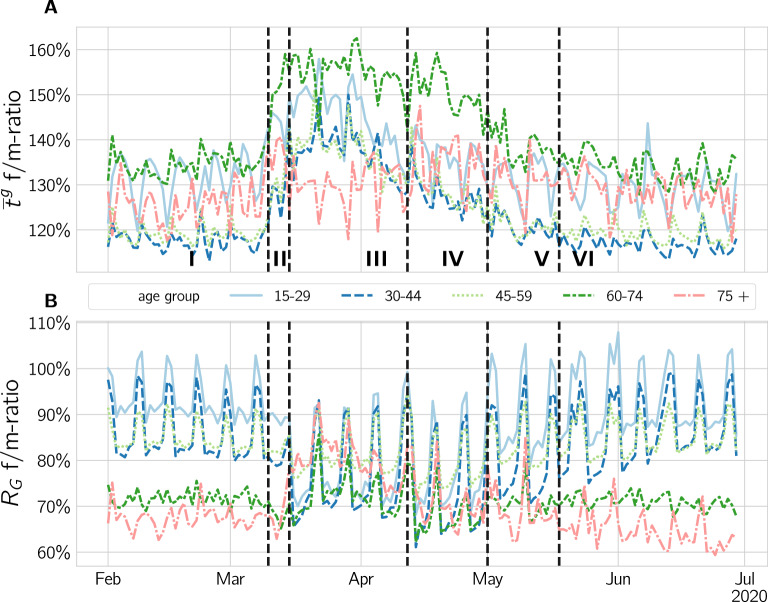
Gender ratios of communication and mobility for different age cohorts. The gender ratio of (**A**) the median call duration, $\bar{t}$, and (**B**) the radius of gyration, $R_G$, is seen. In III the $R_G$ gender ratio of young cohorts is shifted towards women moving significantly (p < 0.001) less, while for old cohorts it is shifted towards a more balanced value. In the same period, for all cohorts except 75+, the gender bias for the call duration increases towards women that have a higher call duration.

The age profile for the median call duration is relatively flat for the adult and senior age cohorts and has very low values for the youngest cohort. The call duration increases slightly for the two youngest, but strongly for the two oldest cohorts. For a visualization, see Supplementary Fig. 16. The gender ratio in call duration is biased towards women for all ages during the crisis, as seen in Fig. 3 A. Notably, the age cohort 15-29 is the only cohort having a more balanced call duration on weekends. For all other cohorts gender differences are increased on weekends. Around the beginning of phase III, the ratios for all except the 75+ cohort reach a maximum. The 75+ cohort reaches a maximum of the gender imbalance in phase IV.

In Fig. 2 B we show the number of calls, $N^g_c$, for male and female generated calls. Here we display the mean of $N^g_c$ because the median due to its discrete nature in combination with the relatively small average $N^g_c$ between 3.5 and 4.5, would make changes and gender differences hard to see. After a short increase in calls in phase II (female: +13%, male +6%) we see a significant drop in calls in phase III (both -9%), which never reaches pre-lock-down levels in the observation period. It stabilizes at a level of -5% and -4% of the previous level for women and men, respectively. There are only small gender difference in the number of calls. For a discussion see SI Text S5.

In Fig. 2 C we show the timeseries for the number of different communication partners, $k^g$, i.e. the degree of men and women in their communication networks. For the same reason as for $N^g_c$, we show the average instead of the median for $k^g$. After a brief rise (up to 8% and 13% for men and women, respectively) in phase II, $k^g$ falls below its pre-crisis level ($-3$% and $-2$%). In phases IV and V $k^g$ rises to values higher than the initial values in phase I. In phase VI $k_g$ is about 4% higher for men and 2.5% higher for women.

During normal times (phase I) we find that men have a slightly higher average degree (communication partners) on weekdays (f/m ratio 95%, men 1.6, women 1.55 unique contacts per day), while on weekends it is more or less balanced (women and men 1.4). In phase II, $k_g$ is increased for both genders to a maximum around 1.73, with an increasingly smaller gender bias. In phase III the degree drops below pre-crisis levels, but men reduce $k^g$ stronger, resulting in a smaller gender divide in phase III (96%). From phase IV onward, the degree slightly increases (even above pre-crisis levels: men 1.7 and women 1.6), even stronger for men, hence resulting in an increased gender divide (less than 94%). Supplementary Fig. 15 C shows the age dependence of the gender ratio for the degree. Again, there is a weekend trend towards women. They have more communication partners on weekends, except for the 15–29 age cohort. The gender ratio increases in phase III for all age cohorts.

Call duration increases much more than the number of calls decreases, regardless of gender. This is visible in Fig. 1 D and E. Just in phase II there is a drastic rise in both, call time per call, and the number of calls. The concentration of communication partners is higher for females and increases during crisis. The bias is also shifted towards men having more communication partners in phase VI. All proxies indicate a strengthening of individual contacts and a focus on important contacts.

Gender ratios of different phases are considered to be distributed around different stationary values. Subsequently, we compare them with a two-sided Mann-Whitney-U test, and reject the null hypothesis that they are from the same distribution. The results of the significance tests are presented in SI Text S6, for all age groups, separated into weekdays and weekends. In SI Text S7 we present additional information on the robustness of our results with respect to geographical heterogeneity and different quartiles of the distribution.

### Mobility

**Figure 4:**
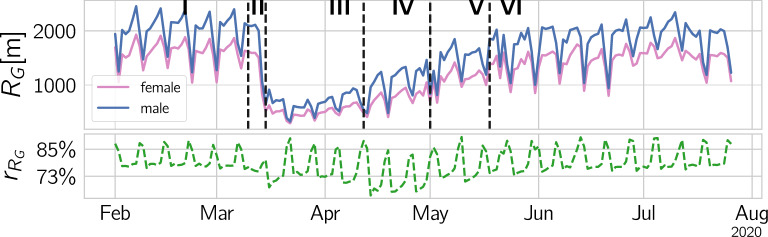
Mobility quantified by $R_G$. The upper panel shows $R_G$ for men (blue) and women (pink). The lower panel depicts the gender ratio, $r_{R_G}$, over time. We observe a large drop in $R_G$ for both genders in phase III and a drop in gender ratio in phases III (lock-down), IV, and V (lock-down eased).

In Fig. 1 C we see the overall decline of mobility in terms of the radius of gyration, $R_G$. Austrians move drastically less during the lock-down, start to move again when the first easing occurs in mid April, and return back to normal in phases V and VI. Figure 4 A shows $R_G$ for the two genders, $R^f_G$ (red) and $R^m_G$ (blue). The gender-ratio, defined as $r_{R_G} = R^f_G / R^m_G$ is depicted in panel B. The female population is moving less than males in pre-crisis times (phase I), as seen in the ratio $r_{R_G}$ of 78% on weekdays and 88% on weekends. After a brief transition period II the weekday ratio drops to around 73% during the lock-down phase III, while on weekends the ratio remains at initial levels. In phase IV, once restrictions were lifted, $R_G$ for males returns back to normal more quickly than for females, hence decreasing the gender ratio further down to 67%. The ratio starts to recover towards pre-crisis levels starting from phase V onward, once the main restrictions were lifted. When fitting the $R_G$ curves as they converge to pre-crisis levels after the lock-down, we report a *half-life time* for men of $t^m_{1/2} = 34.8$d, and $t^f_{1/2} = 36.0$d for women. For details of the fitting, see SI Text S2.

The changes in gender ratios of $R_G$ are significant between the phases. For the significance tests, see Supplementary Tab. 5 in SI Text S6. Especially the changes from phase I to the subsequent phases and from III to phase IV are indeed highly significantly. We find similar results if we replace the radius of gyration by an alternative measure for mobility that is inspired by entropy, $S^{f/m}_i$. It is presented and discussed in SI Text S8.

In Fig. 3 B we show the age-stratification of the gender-ratios. Before the crisis we observe very different gender ratios for different ages. Generally the ratio decreases with increasing age. For the young cohort of 15–29 years, the weekday-ratio is above 90%. For the two age cohorts above the average age of first childbirth (26.3 years for women and 28.7 for men^39^), 30–44 and 45–59, the ratio is reduced to about 83%. For the age cohorts of retirement, 60–74 and 75+, gender disparity becomes even more biased towards men with a ratio of about 70%. In phase III, the three younger cohorts show an overall trend of increasing gender biases. For the age cohort 45–59, this trend is much less pronounced. Strikingly, the effect is reversed for the retirement cohorts where the gender ratio changes from around 70% to more than 80%, which again decreases towards pre-crisis levels in phase IV. The ratio for the old cohorts returns much more quickly to pre-crisis values than all the younger ones, which do not return to the previous values until the end of the observation period. We do not observe large differences in half-life times across gender, but $t_{1/2}$ is much smaller for older cohorts. For all cohorts we find values between $t_{1/2} = 38.8$d for 15-29 year old women to $t_{1/2} = 28.8$d for 75+ year old men. For more details, see SI Text S2. For the corresponding statistical tests, see SI Text S6.

The radius of gyration can be compared with corresponding data of the previous year (2019) in the same time period. We find that during the lock-down phase in 2020, there is less than 40% of the movement than in 2019, see SI Text S8. We show additional information on the statistical, geographical, and temporal robustness of our results in SI Text S7. We provide the timeseries for the quartiles of $R_G$ in Supplementary Fig. 19, the distribution of $R_G$ gender ratios across political districts in Supplementary Fig. 6, and data on the second lock-down in the autumn of 2020 in Supplementary Fig. 23.

### Basic provisioning

In Supplementary Fig. 26 A we show the number of unique devices as a proxy for the number of people at a shopping center across the lock-down. We count the number of unique subscribers in a specifically defined area, see SI Text S3 for the exact definition. In Supplementary Fig. 26 B the corresponding gender ratio is shown. The shopping center is the largest of its kind in Austria and one of the largest in Europe. It is a cluster of 359 shops spread over an area of 670,000 m$^2$. Shops sell a wide range of products, including sports equipment, garments, furniture and electronics. It is visited by more than 20 million visitors each year from Vienna and its hinterland, especially in the south, as well as from Hungary and Slovakia. There are also 14 shops, including supermarkets, drug stores and pharmacies that were not affected by the lock-down.

The visiting patterns of the shopping center in phase I show a pronounced weekly periodicity with a maximum on saturdays and very few visitors on sundays, when all stores except cinemas and restaurants are closed. The gender ratio in phase I is close to one, indicating gender balance. In phase III the shopping complex was shut down to a large extent. No businesses other than stores for basic provisioning were allowed to open. Nevertheless we find a small number of visitors that we account mainly to persons shopping for food and drugs. The gender ratio in phases III and IV is clearly male-dominated (see Supplementary Fig. 26 B and for p-values, see SI Tab. 7 in SI Text S11). In phase V, when shops were allowed to re-open, visitor numbers rose to pre-crisis levels at the beginning of the week, however without the strong peaks on Saturdays. The gender ratio returns to a balanced situation; compare with SI Tab. 7. For a comparison with the same period in 2019 we refer to SI Text S10.

### Leisure activities

In Supplementary Fig. 28 A we count the numbers in a popular recreational area nearby Vienna, the Kahlenberg, frequented mainly for walks, and easy hikes. The number of visitors does not drop in phases II–V, but increases with the usual seasonal trend from march to june. For a comparison with the situation in the year 2019, see SI Text S12. We find more visitors on weekends and on days with good weather, explaining the high variance in numbers. The gender ratio is biased towards women during phase I, which changes in phase III, where we find a more balanced gender ratio. Interestingly, the gender ratio does not return to pre-crisis values after the lock-down, see Supplementary Fig. 28 B. The corresponding statistical tests are found in SI Tab. 7 in SI Text S11.

### Circadian rhythms

**Figure 5:**
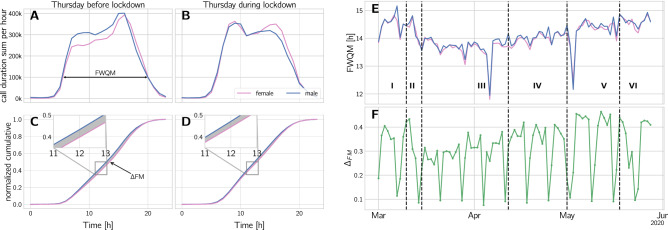
Changes in circadian rhythms during the lock-down measured by the call duration (in s) in the whole network. (**A**) Phone network traffic measured by call duration per hour on the last thursday in phase I, March $4^{\mathrm{th}}$. The horizontal arrow marks the full-width-quarter-maximum length (FWQM). (**B**) Call duration per hour on thursday, march 18th, the first thursday in phase III. (**C**) Normalized cumulative activity for the day shown in panel A. The inset highlights the difference of the male and female curve. The gray shaded area marks the difference between the circadian rhythm of men and women, denoted by $\Delta _{FM}$. (**D**) Same as in C, but for the curve in panel B. (**E**) FWQM for men and women over time. (**F**) As the gender ratio of FWQM does not change significantly, we show the gender difference in circadian rhythm, $\Delta _{FM}$, over time. For both genders, the activity maximum shifts from late afternoon to morning and the length of the activity period is approximately 45 min shorter during the lock-down. A reduction in $\Delta _{FM}$ means that circadian rhythms of men and women become more synchronized.

We compare aggregated phone network traffic across the 24 h of a day for women and men, to estimate gender differences in circadian rhythms. For definitions, see SI Text S3. Figure 5 A and B show the call time per hour for the last wednesday in phase I and the first wednesday during the lock-down. The maximum activity shifts from the late afternoon to the morning hours. The average full-width-quarter-maximum (FWQM) captures the length of the daily activity period. We find that the FWQM is reduced by approximately 53 min from $14 \text {h} 33 \text {min}$ in phase I to $13 \text {h} 40 \text {min}$ in phase III. The results are displayed in Figure 5 E. We do not find a significant change in the gender ratio of the activity FWQM (Mann-Whitney U, $p>0.05$).

To capture the shift of the activity of men and women to different times of the day, we calculate the normalized cumulative functions of the call duration, as shown in Fig. 5 C and D, thereby correcting for different total activity. Following^22^ we compare gender differences by calculating the area between the curves $\Delta _{FM}$, see Fig. 5 C by the gray shaded area, see Methods in SI Text S3. A large (small) value indicates that the activity of men and women takes place at different (the same) times of day. As shown in Figure 5 F, we find that $\Delta _{FM}$ reduces by 25% from phase I to phase III. The values for FWQM and $\Delta _{FM}$ across the crisis are shown in panels E and F. The significance of these findings is again shown with a two-sided Mann-Whitney U test that rejects the null-hypothesis that the values are drawn from the same distribution with $p < 3 \times 10^{-5}$. It confirms that $\Delta _{FM}$ is indeed lower in phase III.

Qualitatively we find the same behavior for the sum of gigabytes up- and downloaded and the number of calls. The reductions in lengths of day range from 40 min for the downloaded gigabytes to 60 min for the number of calls. For the corresponding analyses, see SI Text S13.

## Discussion

The COVID-19 pandemic represents a unique natural experiment to understand individual and collective coping mechanisms with respect to stress and crisis. Telecommunication data reveals almost real-time insights into many aspects of daily life without interfering with the subjects’ actions and interactions. Using anonymized mobile phone data of a large fraction of the Austrian population, we find that gender differences that can be observed in communication patterns, mobility, and spending leisure time are amplified during the crisis, imposed by a severe lock-down in the first phase of the COVID-19 crisis. In the context of basic provisioning, we find indications that during the crisis there exists a bias toward men doing the shopping for food that is absent in normal times. Circadian rhythms change such that for both, men and women, daily activity is concentrated more on a shorter period of the day. Circadian rhythms of men and women tend to be more synchronized during the lock-down.

For both genders we observe an increase of total call duration, which is due to an increase of the call time per call and, interestingly, a decrease in the number of calls. This is a clear sign that communication becomes more focused and intense. This finding is in line with a general decline of the number of communication partners during the lock-down, suggesting a focus on a core of communication partners. The reduction of communication partners could result from the loss of conversation partners from work, however, we also observe a reduction on weekends, where one would not expect effects from professional contacts. The degree distribution before the crisis is in line with earlier work on mobile phone data^40^. While they find a mean degree 2.34 (averaged over 18 months), we get a smaller value of 1.53, presumably, because we average over 24 h. However, we find the same power-law exponent, $\sim -8$, for the degree distribution. In these quantities we see a clear increase and amplification of the gender-biases.

Women show a smaller decrease in the number of calls and a stronger increase in call time per call. As a consequence, the gender ratios of the respective quantities shift towards females. Women have been reported to have more tightly knit (online) networks than men^8, 41^. We interpret our findings as a signal that this behavior intensifies during crisis. The tightening of the social network can also be attributed to social carework, such as calling lonely elderly, which was reportedly performed more often by women during the lock-down^42^. In previous studies, women were reported to employ more active, problem-oriented coping strategies such as emotional and social support, while men show rational and detachment strategies in response to everyday stress^5^ and during a community crisis^4^. This, again, supports the expectation that women seem to tighten their social networks more than men.

We find that the recovery time to women’s total call time initially is as fast as for men, but later, clearly slows down. The increase of demand for communication can be interpreted in the context with higher needs for communication as a coping strategy in an ongoing crisis^4, 5^. It also aligns well with the fact that women experience more stress than men^43^, have higher levels of post traumatic stress disorder^44^, and have a higher prevalence to depression, partly due to “stress responsiveness”^45^. For the COVID-19 pandemic, similar results have been reported. For example, a study in Spain found that women showed more symptoms of depression, anxiety and PTSD, more feelings of loneliness, and less spiritual well-being when compared to men^46^. Our result could be confounded by gender differences introduced by work environments. However, increasing gender-ratios in call times per call and the number of calls on weekends are indicators that the confounder indeed weakens the effect on weekdays.

The age stratification of call times and the number of calls seemingly suggest that younger cohorts communicate less than older ones. We attribute this to a higher proportion of instant messaging services^47^ and other modern communication channels in the younger cohorts. Here a channel selection bias towards younger cohorts using web-based communication services more actively acts as a severe confounding factor.

The female population is moving less over the entire period, confirming earlier work in different countries and contexts^2, 38^. The decrease in mobility, following the lock-down is stronger for women. In addition, men recover their mobility behavior much more quickly after the measures are lifted. This effect depends on age. For the young and adolescent population the existing gender-bias in mobility is enhanced, while for those above retirement age the bias reduces. We relate this to childcare duties during the reproductive age and gender specific differences in occupation. Unequal distribution of childcare work has been a large concern at the beginning of the pandemic^30–32^. Several studies identified it as a driver of gender inequality^48, 49^. Our data supports this hypothesis as the gender ratio is significantly (MWU p<0.0001, see SI Text S6) more equal after the school openings in phase VI. Occupational differences become apparent in the unemployment numbers at the beginning of phase III, where the increase for women was 8.7% larger than for men (women +67,5%, men 58,8%)^27^.

In addition to care-taking duties and occupational differences, the literature suggests an additional effect: Women have been shown to exhibit more ethical behavior, at least where it is socially desirable, while men often behave less community-aware^50, 51^. For women, it has been shown that they are 50% more likely to adopt non-pharmaceutical interventions in response to a respiratory epidemic^52^. In this context, the reduction of mobility in women could be partly attributed to responsible behavior in staying at home to protect vulnerable parts of the population. This argument is supported by a qualitative panel survey, that reports women taking the COVID-19 pandemic more seriously in Austria^53, 54^.

Since it seems that men move more for work-related issues and are more often responsible for gathering basic provisions during the lock-down, they are more exposed to the perceived danger of catching SARS-CoV-2. One could speculate that this might be a sign of higher risk-taking behavior in men, in line with several previous arguments^8, 55, 56^. For a conclusive clarification of this matter, obviously, more research is needed.

Generally, gender differences in mobility decrease on weekends. We confirmed that the radius of gyration is larger for men because they commute more/farther^2^. This suggests that a main factor for our observed behavioral changes is indeed employment. Further evidence for this hypothesis is found in the fact that only for the 60+ age cohort the gender-ratio does not change between weekends and weekdays. Nevertheless, the effects discussed above persist on weekends and our conclusions remain valid.

We approximate the activity of people by analyzing their Internet traffic loads across a day. On average, the daily activity period is reduced by the lock-down by about 40–60 min. The gender-ratio of the effective length of a day does not change significantly. However, mobile phone usage of men and women occurs at different times of the day, with the maximum shifted from the late afternoon to the morning during the lock-down. A study that combines questionnaires and mobile phone communication of 24 young adults during the transition phase of highschool to work (or university)^57^ finds that even though there is a strong turnover in the social network, the times of day at which calls happen are highly personal and persistent over time. It is intriguing that we observe an aggregate shift away from the pronounced activity maximum in the late afternoon, also found in^57^, towards a maximum in the morning. To disentangle the individual characteristics from a social synchronization phenomenon that causes this shift could be a fruitful direction of future research.

Network traffic starts to increase later in the day and ends earlier. This can be explained by commutes becoming obsolete because of home office and the rise in unemployment^30–32^. We believe that the shift of the maximum activity from evening to morning is caused, on one hand, by different activity patterns in home office, and on the other hand, by different spare time activities during the lock-down.

The circadian rhythms of women and men become more synchronized during the lock-down, likely as a consequence of people staying at home much more than usual, where often persons of different gender are present^39^. It would be interesting to understand if synchronization is stronger for inhabitants of the same household. More detailed studies of the effects of the pandemic on the circadian rhythm, with respect to age and personal attributes, such as morningness or eveningness^58^, could yield more detailed insights in, e.g., disturbed sleep patterns.

We have shown that a massive collective crisis will result in tighter social networks with a focus on a social core environment. Women seem to focus more on this tightening, indicating stronger, more active coping strategies, a different perception of the dangers, and a stronger pro-social behavior. We see that mobility is reduced much more for females, and that their time to recovery is considerably slower. This is partly work-related and maybe associated to a stronger community-aware behavior in response to public mobility restrictions. We see a slight indication of increased risk taking in males when it comes to basic provisioning. Finally, we report synchronization effects of (online) activity behavior during the day between males and females during crisis.

Our study uses a massive data set to analyze the digital traces of 1.2 million Austrians, allowing us to provide quantitative empirical data on gender differences in Austria during the COVID-19 pandemic. To complement our results we tried to contextualize them with the results of previous qualitative analyzes highlighting psychological, social, and economic reasons for the observed differences.

## Methods

### Data

We partnered with a large Austrian internet service provider (ISP) to get access to data from mobile phones. We use a combination of classical Call Data Records for the voice domain as well as a combination of generic data records (known as X Data Records) for the data domain. Thus we do not only register an event when a call is performed, but rather perceive additional events when data packages are transferred. Various network interfaces are connected via probes so we get data points from a multitude of network technologies for mobile data usage (2G, 3G, 4G), calls, text messages as well as Voice over LTE, from both user- as well as control plane. On average we observe approximately 1 Billion events per day, 4.5 Million devices per day and for 80% of the devices the next event is received in 1.7 min, on average 4 min. When evaluating gender differences we need to filter the data to approx 1.2 Million devices per day where demographic details are defined. Demographic information is not available for roamers or virtual mobile operators (MVNO) and thus they are excluded from this analysis. Further, only devices with a radius of gyration $R_G$ (see eq. (1) below) larger than 0m and lower than 300km are considered. The lower bound aims to exclude internet of things-devices, which typically do not move, such as LTE-internet routers. The upper bound excludes a small number of devices which have a $R_G$ larger than the theoretically maximal $R_G$ inside Austria and are attributed to network artifacts. Calls are filtered to a length of at least 25 seconds prior to aggregation to exclude calls that were not picked up, which form a distinct peak just below 25 seconds, shown in Supplementary Fig. 1.

As the gender attribute in the data set is self-reported and we discuss psychological, social and biological reasons for the observed behavioral changes, we will use the term ‘gender’, referring to the psycho-social construct.

Supplementary Table 1 outlines the distribution of the devices per cohort and compares them to census numbers. As not all devices are active every day we give the mean and standard deviation as an approximation for the overall counts. Furthermore, in Supplementary Fig. 2 we show the time series of the absolute numbers of devices and in Supplementary Fig. 3 we show the corresponding gender ratio. We show that the geographical distribution is relatively even by comparing the devices per district with census numbers in SI Text 2 and Supplementary Fig. 4. As we cannot analyze a device for more than 24 h (see below), we need to calculate aggregate statistics over the analyzed time period.

Our localization methodology is based on the topology of the network, namely the observed cell-id. This means that the accuracy is limited, and much less accurate than GPS based localization or the result of custom apps combining Bluetooth, WiFi and GPS. However, the data is available for a large quantity of devices. The ISP provides us with the localization information for each cell-id, which is based on the centroid of the network coverage simulation. In SI Text S2 we discuss potential biases arising from different signalling rates.

The data is anonymized, any identifiers are hashed every 24 h with a changing key by the ISP prior to making the data available for the researchers. Only cell-id based localization is used to enhance the privacy of the subscribers and only aggregate and k-anonymized statistics are reported. With this procedure we adhere to the recommendations of the GSMA^59^ with regards to data privacy handling as well as the law of the national jurisdiction.

### Metrics

#### Communication

 By analyzing calls, social interactions can be modeled. This part of the data consists of a list of outgoing (MO) and incoming (MT) calls, each associated with a source and destination. We filter to calls with a duration of at least 25 seconds to adjust for a shift in the distribution corresponding to calls that were not answered (see Supplementary Fig. 1).

For each device we find $N_c^{\mathrm {MO}}$ outgoing and $N_c^{\mathrm {MT}}$ incoming calls with $k^{\mathrm {MO}}$ and $k^{\mathrm {MT}}$ other individuals, respectively (in- and out-degree). The call duration is denoted by $\bar{t}$. Additionally, as described earlier for the mobility dimension, for each device, age group and gender are specified.

For all of these device-level metrics we report the median of the whole population, or for cohorts specified by age groups or gender. We will add superscripts *g* and *h* to indicate gender.

#### Mobility

 We obtain mobility data as a stream of spatially localized network signaling events. It is transformed into a list of locations $\mathbf {x}_{i\mu } = (x_{i\mu }, y_{i\mu })$, with associated stay duration $t_{i\mu }$ for every individual $i = 1 \ldots N_{indiv}$ at location index $\mu = 1 \ldots N_{locations}$, where *x* and *y* represent longitude and latitude, respectively. Due to the anonymization procedure the location index $\mu$ is reset every day and the individual index *i* is reshuffled accordingly. For the individuals, metadata is collected in a vector $m_i = (g_i, a_i)$, containing gender $g_i \in {female, male}$ and age $a_i$ aggregated into cohorts of 15 years.

The *radius of gyration* $R_G$ is calculated as the square root of the time-weighted mean of the squared distances *d* (Calculated as the Haversine distance which calculates a distance in meters from latitude and longitude coordinates given in degrees.) of the locations $\mathbf {x}_{i\mu }$ to the daily centroid $\overline{x}_i = \frac{\sum _\mu \mathbf {x}_{i\mu } t_{i\mu }}{\sum _\mu t_{i\mu }}$:

1 $$\begin{aligned} R_{\mathrm {G},i} = \sqrt{\frac{\sum _\tau d(\overline{x}_i, \mathbf {x}_{i\tau })^2}{\sum _\tau t_{i\tau }}} \end{aligned}$$

It captures the amount of movement in a time weighted manner and has the dimension of a length in meters. The distribution of $R_{\mathrm {G},i}$ is fat tailed, see Supplementary Fig. 6. In the main paper we report the median because it robust to heavy tails.

For our second mobility measure *entropy* the locations $\mathbf {x}_{i\mu }$ are binned into a hexagonal raster using Uber’s H3^60^. The chosen resolution level for the raster yields hexagons with an area of approximately $800m^2$ (This is H3’s resolution level 8.). For each hexagon $\tilde{x}_\nu$ ($\nu = 1\ldots N_{hex}$), the stay duration of the locations in each hexagon are aggregated to $\tilde{t}_{i\nu }$

2 $$\begin{aligned} \tilde{t}_{i\nu } = \sum _{\nu \forall \mathbf {x}_{i\nu } \in \tilde{x}_\nu } t_{i\nu } \end{aligned}$$

The stay time distribution of an individual *i* is then defined as the share of its time spent in a given hexagon $\tilde{x}_\nu$

3 $$\begin{aligned} p(\tilde{x}_{i\nu }) = \frac{\tilde{t}_{i\nu }}{\sum _{\nu }\tilde{t}_{i\nu }} \end{aligned}$$

The entropy of an individual’s stay time distribution, $S_i$, is defined, using the standard formulation of Shannon Entropy, as:

4 $$\begin{aligned} S_i = - \sum _\nu p(\tilde{x}_{i\nu })\log _{2}(p(\tilde{x}_{i\nu })) \end{aligned}$$

#### Points of interest (Shopping, Leisure)

 Specific points of interest reflecting shopping and leisure zones in Vienna were analyzed in more detail. We first used H3 by Uber^60^ to create a discrete grid for the whole country to speed up the analysis of specific locations afterwards. Then we count the number of unique subscribers in a set of manually defined hexagons. We limit our investigations to stays longer than 10 min and shorter than 4 h. We assume this eliminates devices passing the shopping complex on a nearby highway, as well as persons working there, because these activities take much shorter or longer, respectively.

#### Circadian rhythm

 We investigate the circadian rhythm using network traffic measures *A*(*t*) aggregated by gender and ranging from the sum of call duration per hour to downloaded gigabytes per hour. Irrespective of the quantity, we observe a broadened, peak-like structure with a rise in the morning and a drop in the evening. We quantify the duration by the *full-width-quarter-maximum* distance (FWQM). It denotes the time span between point where the activity is larger than the quarter of the maximum activity in the morning and the point where the activity drops below the same value in the evening. We choose the threshold relative to the maximum, so we are independent of the total activity; its value is set to a quarter without loss of generality,

5 $$\begin{aligned} A(t)- max(A(t))/4= 0 \quad \forall t_1, t_2 \end{aligned}$$

6 $$\begin{aligned} \mathrm {FWQM}= t_2 - t_1 \end{aligned} \quad .$$

Male and female activity, corrected for the difference in total activity, is not distributed across the 24 h of a day in the same way. Inspired by^22^ we apply the following procedure. We correct for the difference in total activity by calculating and normalizing the cumulative activity

7 $$\begin{aligned} C(t) = \frac{\int _0^t A(\tau ) d\tau }{\int _0^{24} A(\tau ) d\tau } \quad \text {,} \end{aligned}$$

where 0 and 24 are the time at the beginning and end of the chosen 24 h period. Now we calculate the gender difference $\Delta _{FM}$ in circadian rhythm by calculating the absolute area between the cumulative activity functions for men and women

8 $$\begin{aligned} \Delta _{FM} = \int _0^{t_{max}} | C_m(t) - C_f(t) | dt \end{aligned} \quad .$$

#### Gender differences

 To investigate gender differences we calculate the gender ratio $r_x$ for the various aggregations *x* presented in this work. The ratio $r_x$ is calculated as the quotient of the aggregate for the female cohort divided by the aggregate for the male cohort $r_x = x_{\mathrm {female}} / x_{\mathrm {male}}$ (*x* represents the aggregation, e.g. median $R_G$ or median call duration $\bar{t}$). A gender ratio $r_x$ close to 1 (or 100%) indicates that the quantity is of similar size for both genders, less (more) than 100% indicates smaller (larger) values for females.

### Participants

Only anonymized data was processed for this study and no participants were involved.

## Supplementary Information

Supplementary Information.

## Data Availability

The data used in this study were provided by a large Austrian telecommunications provider. No administrative permissions were required. Legal restrictions apply to the availability of these data, which were used under special agreements for the current study, and so are not publicly available. Data are however available from the authors upon reasonable request and with permission of the anonymous data provider. Source data for the main figures are provided with the paper.
