# Supplementary material for: Behavioral gender differences are reinforced during the COVID-19 crisis
Source: Sci Rep. 2021 Sep 28;11:19241. doi: 10.1038/s41598-021-97394-1 (PMC8478918; doi:10.1038/s41598-021-97394-1)
Supplement: Supplementary file 1 — Supplementary Information. [file 41598_2021_97394_MOESM1_ESM.pdf]

Supplementary Information for “Behavioral gender differences are reinforced during the COVID-19 crisis”

Tobias Reisch[1,2,*], Georg Heiler[2,3,*], Jan Hurt[2], Peter Klimek[1,2], Allan Hanbury[2,3], and Stefan Thurner[1,2,4,+]

[1]Section for Science of Complex Systems, Center for Medical Statistics, Informatics and Intelligent Systems, Medical University of Vienna, A-1090 Vienna, Austria
[2]Complexity Science Hub Vienna, A-1080 Vienna, Austria
[3]Institute of Information Systems Engineering, TU Wien, A-1040 Vienna, Austria
[4]Santa Fe Institute, Santa Fe, NM 85701, USA
[*]these authors contributed equally to this work
[+]stefan.thurner@meduniwien.ac.at

SI Text S1: Partitioning of observation interval

The observation period ranges from Feb 1 to June 29 2020. Our analyses compare various periods of time before, during and after the lock-down. Each period is based on the introduction or easing of one or more non pharmaceutical interventions (NPIs) and identified by roman numerals. Period I refers to the phase before the Austrian population was widely aware of the COVID-19 disease in Austria, and ends with the first press conference announcing public restrictions such as school closings and a call for home office on March 12th.This was followed by a transition period II until Sunday, March 15, 2020, when an extensive lock-down was announced Including all shops closing and restrictions on public movement and gatherings, such as public transport only being allowed to be used for commuting, a ban on meetings with non-household persons and leaving the home only permitted for work that cannot be postponed, grocery-shopping, helping people and short walks. The following lock-down III lasted until Easter (April 13, 2020) which marks the first easing of the measures with small shops and construction stores being allowed to re-open. Phase IV continues until the beginning of phase V on May 1, where the lock-down was eased further and gatherings of up to 10 people were allowed. May 15th marks the beginning of phase VI, with restaurants and bars, as well as schools re-opening. The dates and description of the measures is based on[1].

SI Text S2: Data

We analyze the consistency of the underlying data with respect to the shape of the underlying distributions, the sample composition and the geographic uniformity.

Supplementary Figure 1 shows the histogram for the call duration. At just below 25 seconds there is a small peak, corresponding to calls that were not picked up.

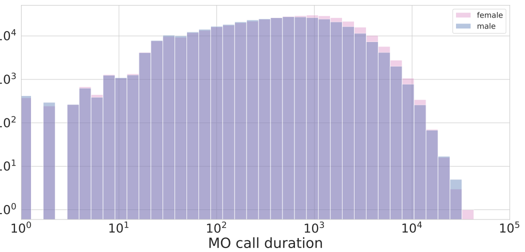

Figure 1. Histogram of the call duration. There is a local maximum around 25 seconds, corresponding to calls not answered, which we exclude by introducing a cutoff at 25s. Note the double logarithmic axes.

As the number of registered devices fluctuates, we report the mean and standard deviation of the number of devices per cohort and day present in the data set. To calculate the coverage of the data we divide the mean with official census data[2]. The smallest coverage is for women over 75 with 3.7%, the best coverage is for men aged 54-60 with 15.7%. To analyze the temporal change in sample composition we plot the absolute numbers and the ratio of the number of female devices over the

number of male devices in Supplementary Figure ?? and Supplementary Figure 3, respectively. We observe a slight variation of approximately 1% around weekends. These fluctuations are addressed by separating our analysis to female and male cohorts and calculating the statistics separately for both.

Table 1. Sample sizes of the gendered demographic cohorts. The census column shows the official numbers for 2020 and the coverage is calculated by dividing the mean devices per cohort by the census number.

gender	age group	mean	std	census	data coverage [%]
female	15-29	44318	3038	757127	5.8
female	30-44	130137	9349	882664	14.7
female	45-59	151173	11576	1011738	14.9
female	60-74	80479	6269	738582	10.9
female	75 +	18890	1726	508807	3.7
male	15-29	42023	2553	799900	5.2
male	30-44	116230	7493	899709	12.9
male	45-59	158313	12686	1007604	15.7
male	60-74	84800	7368	669088	12.7
male	75 +	19085	1915	342785	5.6

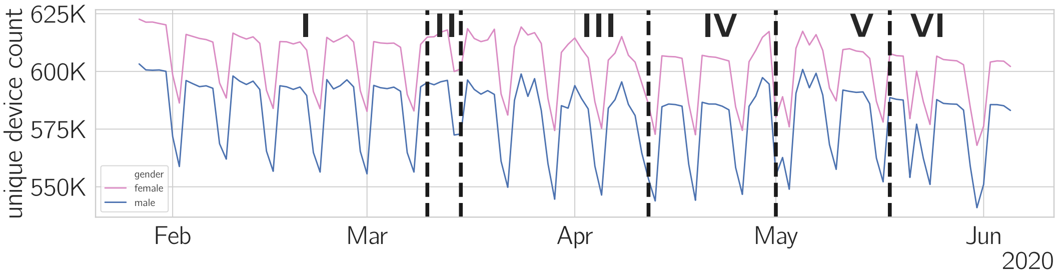

Figure 2. Absolute counts per gender.

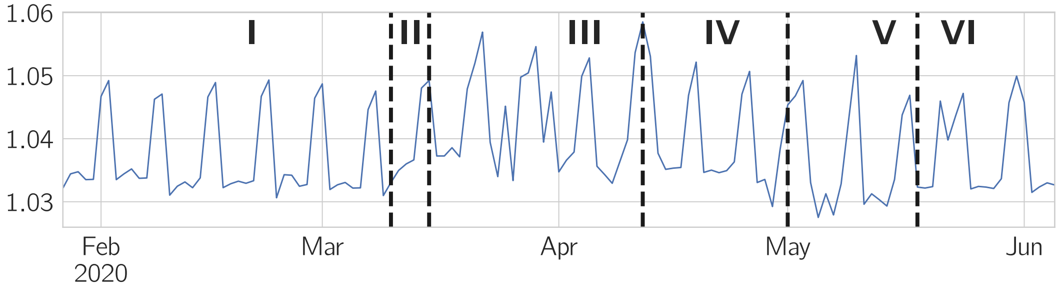

Figure 3. Device ratio

To ensure geographic consistency, we calculate Pearson's correlation coefficient of the number of inhabitants of every political district in Austria with the devices found in that district. We find a value of $r = 0.93$, $p < 10^{-50}$. The corresponding scatter plot is shown in Supplementary Fig. 4.

Another potential data issue is the signalling rate of the used devices. The radius of gyration calculation is more exact for higher signalling rates which are typical for newer devices. In Supplementary Figure 5 we show the gender ratio of the mean signaling rates. Signals are recorded for every change of a cell id or in case the device is static compressed to a keep alive ping every 10 minutes, resulting in a correlation with the amount of mobility. We find a 20-25% lower signalling rate for women. Nevertheless, the weekly pattern stays relatively unaffected across various phases, with up to 3% variation for weekdays and weekends, respectively, leading to the conclusion that the comparisons of the gender ratio of R_G across phases is meaningful.

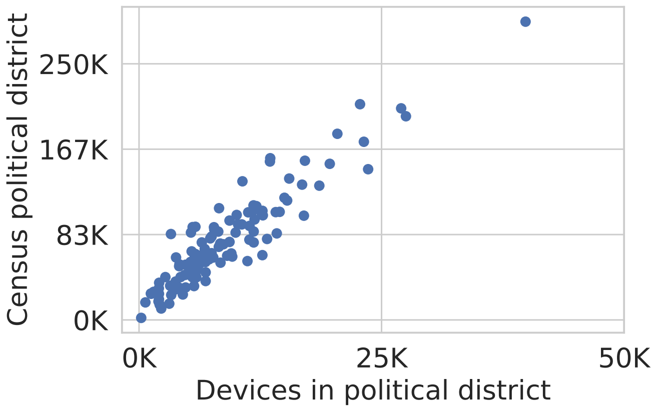

Figure 4. Device ratio

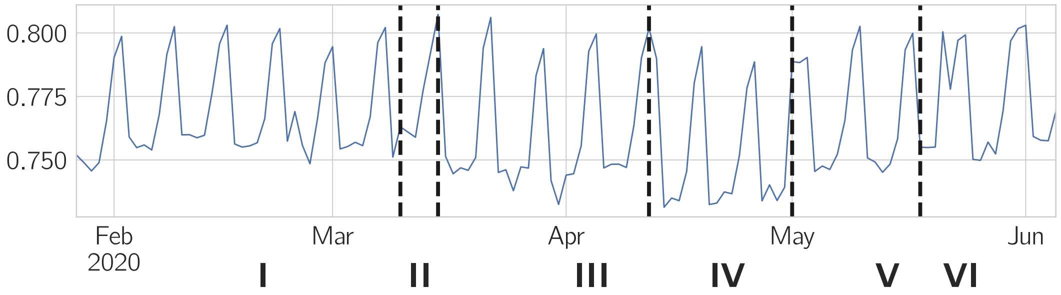

Figure 5. Individual signalling ratio.

SI Text S3: Metrics

Supplementary Figure 6 shows the radius of gyration for one day. It has a fat tail with a cutoff at around 300km, because a larger radius of gyration is not possible within Austria. In the main paper we report the median, as it is robust to fat tails.

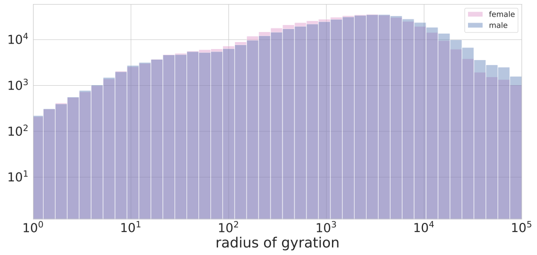

Figure 6. Histogram of the radius of gyration R_G. Note the double logarithmic axes and the truncated fat tail.

SI Text S4: Time-back-to-normal: Fitting Half-life times

For many of the investigated quantities, the COVID-19 lock-down represents a perturbation from an equilibrium with a subsequent, smooth return to the previous value. We fit an exponential function

$$f(t;a_0,a_1,b) = a_0 + a_1 b^t,$$

where a_0 is fixed to the mean value in phase I, a_1 is the additional offset at the beginning of the decay and $b < 1$ is the average daily reduction factor. From b we can calculate the half-life time

$$t_{1/2} = \frac{ln(1/2)}{ln(b)}$$

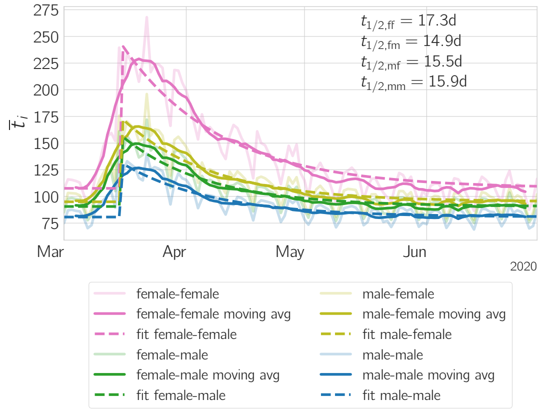

Figure 7. Decay parameters for the call duration $\bar{t}^{gh}$. We show $\bar{t}^{gh}$ (transparent line), the seven day moving average (solid line) and the fitted curve (broken line). The half-life times for the return from perturbed state to normal are 17.3d for male-male, 14.9d for female-male, 15.5d for male-female and 15.9d for male-male interactions. Detailed results of the fitted parameters are in Tab.2.

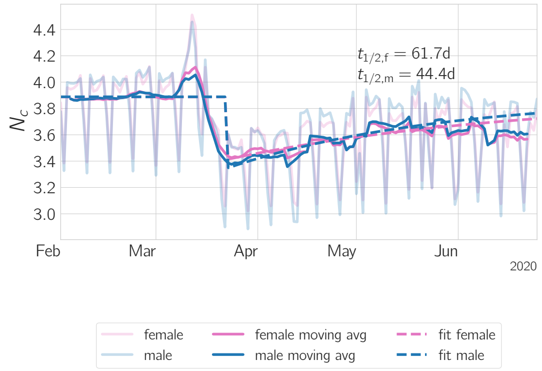

Figure 8. Decay parameters for the number of calls N_c. We show N_c (transparent line), the seven day moving average (solid line) and the fitted curve (broken line). The half-life times for the return from perturbed state to normal are 61.7d for women and 44.4d for men. Detailed results of the fitted parameters are in Tab. 2.

in days, which quantifies the time it takes the fitted quantity to return halfway back to "normal". The non-linear least squares fit is performed to the 7-day moving average of the investigated quantity, starting from the beginning of phase III to the end of the observation period. We report standard deviations σ calculated as the square root of the diagonal elements of the covariance matrix.

Figure 7 shows the average interaction time $\bar{t}^{gh}$, its 7-day moving average and the fitted exponential. We find half-life times between 14.9d and 17.3d for female-male and female-female interactions, respectively. The detailed results are given in Tab. 2. The female-female interaction $t_{1/2}$ is more than one standard deviation larger than for the interactions where males are involved.

We also fit for the number of calls N_c and the number of unique contacts k, see Figs. 8 and 9, and Tab. 2 for details. Both times, the half-life times for females are more than one standard deviation larger. For N_c the half-life times are large compared to the other quantities, and in fact it has not reached the pre-crisis levels by the end of the study period.

Table 2. Fitted parameters for the exponential decay of communication measures. The confidence intervals are one standard deviation.

quantity	gender	a_0	a_1	b	$t_{1/2}$
$\bar{t}_i$	female-female	107	138 ± 4	0.9607 ± 0.0015	17.3 ± 0.7d
$\bar{t}_i$	female-male	91	69 ± 2	0.9546 ± 0.0017	14.9 ± 0.6d
$\bar{t}_i$	male-female	95	81 ± 2	0.9562 ± 0.0017	15.5 ± 0.6d
$\bar{t}_i$	male-male	81	52 ± 1	0.9573 ± 0.0015	15.9 ± 0.6d
N_c	female	3.89	-0.48 ± 0.01	0.9888 ± 0.0010	61.7 ± 5.6d

Table 2. Continuation:

quantity	gender	a_0	a_1	b	$t_{1/2}$
N_c	male	3.89	-0.55 ± 0.02	0.9845 ± 0.0012	44.4 ± 3.5d
k	female	1.524	-0.052 ± 0.007	0.9426 ± 0.0115	11.7 ± 2.4d
k	male	1.582	-0.074 ± 0.014	0.9260 ± 0.0198	9.0 ± 2.5d
R_G	female	1564	-1331 ± 41	0.9809 ± 0.0010	36.0 ± 1.8d
R_G	male	1959	-1606 ± 48	0.9803 ± 0.0009	34.8 ± 1.7d
S_i	female	0.73	-0.53 ± 0.02	0.9761 ± 0.0015	28.7 ± 1.9d
S_i	male	0.78	-0.52 ± 0.02	0.9753 ± 0.0016	27.6 ± 1.8d

We perform the same procedure for the mobility-related quantities radius of gyration R_G and stay-time distribution entropy S_i and show the fits in Figs. 10 and 11. The results for the fits are reported in Tab. 2. The half-life times of females are slightly, but not significantly larger.

For R_G we also stratify for age, see Tab. 3.

Table 3. Stratifying the fitted parameters of the exponential function for age for radius of gyration. The confidence intervals are one standard deviation.

gender	age	a_0	a_1	stdev a_1	b	stdev b	thalbe	thalbe stdev
female	15.0	2318.96	0.9822	0.0009	-1997	63	38.6	2.0
female	30.0	1809.65	0.9810	0.0009	-1564	48	36.2	1.8
female	45.0	1761.09	0.9810	0.0010	-1446	45	36.2	1.8
female	60.0	1038.4	0.9799	0.0010	-860	27	34.2	1.7
female	75.0	526.376	0.9788	0.0011	-346	12	32.3	1.7
male	15.0	2498.53	0.9819	0.0009	-1948	60	38.0	2.0
male	30.0	2126.9	0.9802	0.0010	-1696	51	34.7	1.7
male	45.0	2089.17	0.9808	0.0009	-1663	51	35.7	1.8
male	60.0	1446.69	0.9791	0.0010	-1195	37	32.8	1.6
male	75.0	786.449	0.9762	0.0014	-610	24	28.8	1.7

Through visual inspection it is clear that the quality of the fits of the mobility quantities are not good because the exponential function does not represent the functional form of the return to normal well. The derivative of R_G and S_i is small during the lock-down in phase III and gets larger in subsequent phases when the restrictions are lifted. We fit a logistic function of the form

$$f(t;\alpha,t_0) = \frac{1}{1+e^{-\alpha(t-t_0)}} \quad .$$

The turning point, with the largest derivative, is at t_0 and α controls the time it takes the logistic function to transition from one niveau to the other.

In Fig. 12 we show the results of the results of fitting the step function, the values for t_0 and α are reported in Tab. 4. The α parameter is not significantly different, but the turning point t_0 is significantly larger for females.

The same holds for our second measure of mobility, the entropy S_i. The fits shown in Fig. 13 and values reported in Tab. 4 have not significantly different values for α, but the turning point t_0 for females is significantly later. The extended flat period in the beginning of the logistic function accounts for the extended duration of the lock-down.

Table 4. Fitted parameters for the logistic function. The confidence intervals are one standard deviation.

quantity	gender	α	t_0
R_G	female	$0.0932 \pm 0.0070\text{d}^{-1}$	47.6 ± 0.8d
R_G	male	$0.0901 \pm 0.0078\text{d}^{-1}$	44.3 ± 1.0d
S_i	female	$0.0877 \pm 0.0078\text{d}^{-1}$	46.2 ± 1.0d
S_i	male	$0.0857 \pm 0.0087\text{d}^{-1}$	43.9 ± 1.2d

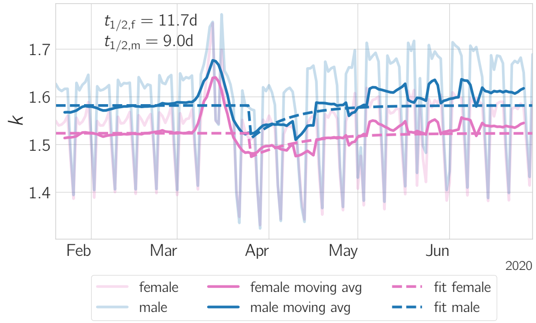

Figure 9. Decay parameters for the number of interaction partners k. We show k (transparent line), the seven day moving average (solid line) and the fitted curve (broken line). The half-life times for the return from perturbed state to normal are 11.7d for women and 9.0d for men. Detailed results of the fitted parameters are in Tab. 2.

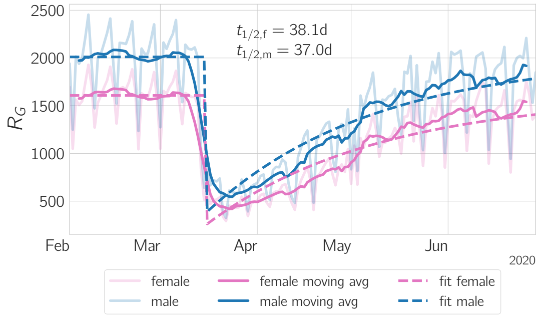

Figure 10. Decay parameters for the radius of gyration R_G. We show R_G (transparent line), the seven day moving average (solid line) and the fitted curve (broken line). The half-life times for the return from perturbed state to normal are 36.0d for women and 34.8d for men. Detailed results of the fitted parameters are in Tab. 2.

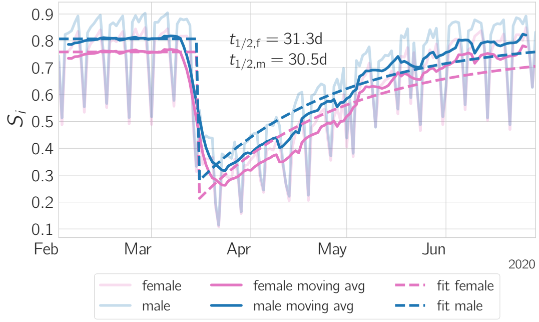

Figure 11. Decay parameters for the stay time entropy S_i. We show S_i (transparent line), the seven day moving average (solid line) and the fitted curve (broken line). The half-life times for the return from perturbed state to normal are 27.6d for women and 28.7d for men. Detailed results of the fitted parameters are in Tab. 2.

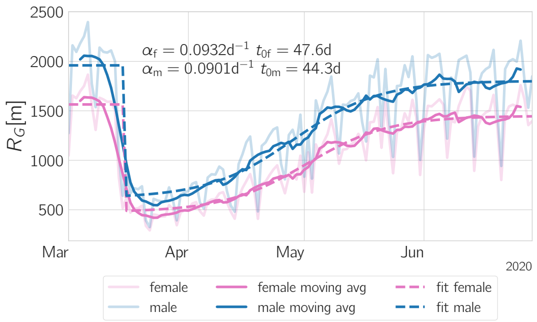

Figure 12. Logistic fit for the radius of gyration R_G. We show R_G (transparent line), the seven day moving average (solid line) and the fitted curve (broken line). The decay rate parameter α is similar for both genders, but the turning point t_0 for men is earlier than for women with 44.3d and 47.6d after beginning of the lock-down. Detailed results of the fitted parameters are in Tab. 4.

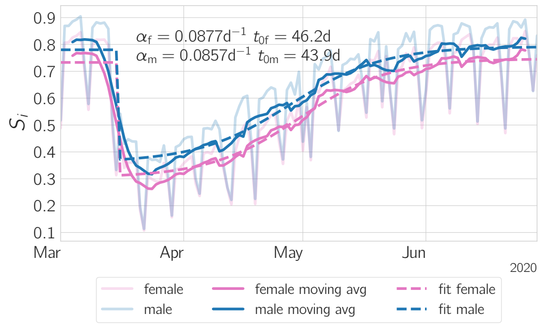

Figure 13. Logistic fit for the stay time entropy S_i. We show S_i (transparent line), the seven day moving average (solid line) and the fitted curve (broken line). The decay rate parameter α is similar for both genders, but as for R_0 the turning point t_0 for men is earlier than for women with 43.9d and 46.2d after beginning of the lock-down. Detailed results of the fitted parameters are in Tab. 4.

SI Text S5: Further discussion of communication quantities

In this SI Text we provide additional results concerning communication patterns.

The absolute values of the interaction time $\bar{t}^{gh}$ are given in the main text Fig. 2. Here we discuss the gender ratio and stratify for age. Supplementary Fig. 14 A shows the call time female initiated over male initiated ratio $r_{\bar{t}}$ across the six phases. It is typically shifted towards women (+12%) and even more so on weekends (+16%). In the transition phase II the relative gender gap approximately doubles to +27% on weekdays and up to +35% on weekends. Subsequently we observe a smooth return to pre-lock-down values.

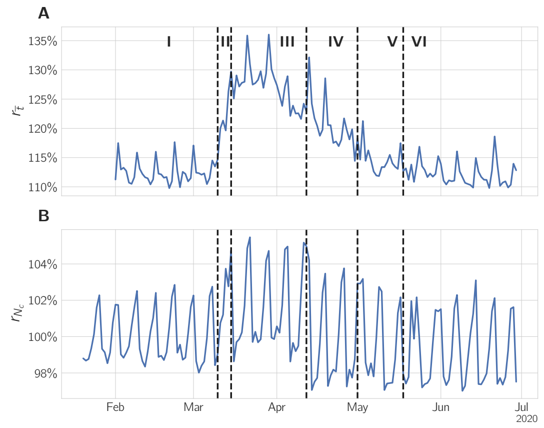

Figure 14. Gender ratios of communication quantities. (**A**) The ratio $r_{\bar{t}}$ of the call time of female over male initiated interactions starts to spike in II, increases in III and recovers subsequently. (**B**) The ratio r_{N_c} of the number of female over male initiated calls is increased during III, but recovers subsequently.

We stratify for age in Fig. 16 A, where we show the average $\bar{t}^g$ over calendar week 10 in phase I and over calendar week 12 in phase III for all age cohorts. The phase I profile is flat with values slightly above 300s for women and slightly below 300s for men. In the lock-down phase III the call time increases for both genders and all ages, but stronger for women and stronger for older age cohorts. The 15-29 cohort increases their communication behavior the least. The gender ratio for $\bar{t}^g$ is shown in Fig. 16 B. It is shifted towards women for all age cohorts and the lowest for ages 30-59. We see a strong increase in week 12 for all ages, except the 75+ cohort. In Fig. 15 A we show the time series of the age ratios, already discussed in the main text.

Next, we discuss the the number of calls as shown in main text Fig. 2 B. As shown in Fig. 14 B, before the crisis women tended to have slightly fewer calls than men on weekdays (3% less) and more on weekends (2% more). This changes in phase III, where the bias shifts towards women having equally as many calls on weekdays as well as an increased bias on weekends (4% more). The bias returns to normal levels in phase IV, however, the weekday ratio is shifted to be more male-biased. We report significance tests in SI Text S6.

The gender ratio of N_c, shown in SI Fig. 15 B, deviates only weakly from equality before the lock-down. All cohorts, except 15-29, are biased towards females having longer calls on weekends, except for the 75+ which does not exhibit a weekly rhythm. In phase III the gender ratio for all age cohorts shifts towards women having more calls.

The age profiles in Fig. 17 elucidate this a bit more, by showing that from week 10 to week 12 the number of calls decreased for all age cohorts and especially for young cohorts, which have a higher initial level in week 10. The gender ratio increases from week 10 to week 12 for all age cohorts, except for the cohort 15-29, which remains at the same value.

Mean degree varies around 1.4 and 1.7, so the range is relatively small. The age stratification in Fig. 18 A shows that degree is highest for the age cohort 30-44. We see that for the age cohorts younger than 45 there is a reduction in degree, for men 45-59 there is a very small reduction and for women older than 45 and men 60+ there is an increase in degree.

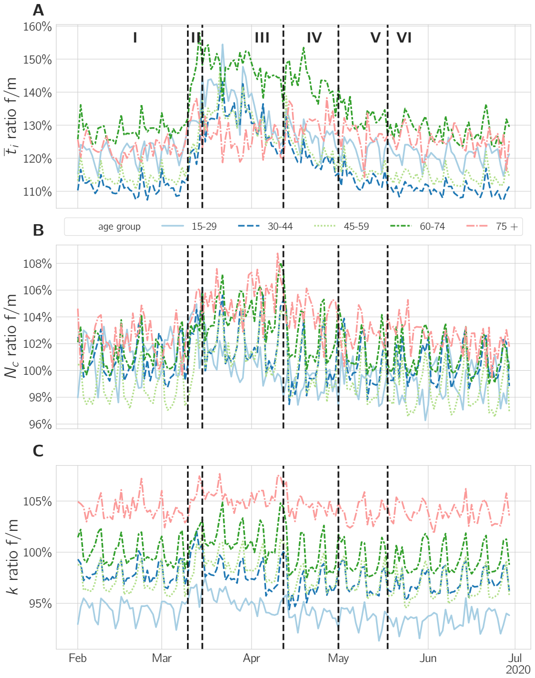

Figure 15. Age dependence of communication strength gender ratios. (**A**) Gender ratio for call time $\bar{t}$, (**B**) gender ratio for number of calls (**C**) gender ratio for degree. The gender ratios overwhelmingly shift towards women communicating more. The effect is the weakest for the oldest cohort. The youngest cohort 15-29 has a different weekday - weekend pattern than the rest of the age cohorts.

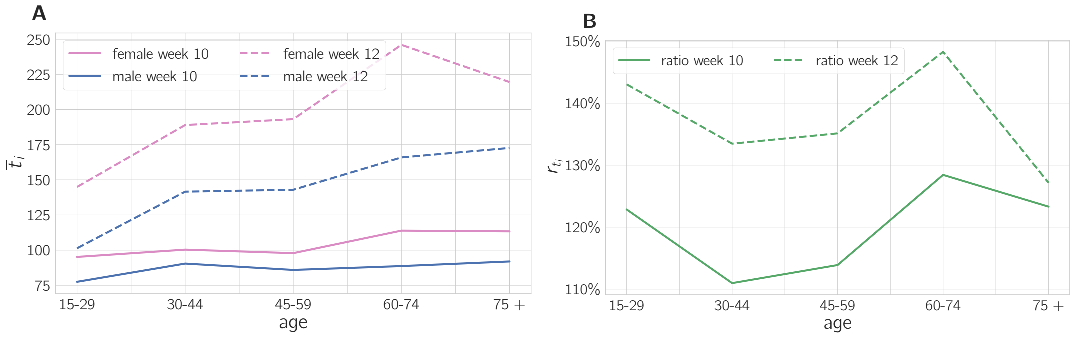

Figure 16. Age profile call duration $\bar{t}$. (**A**) Weekly average in $\bar{t}$ of week 10 (solid line) and week 12 (broken line). (**B**) Gender ratio of the weekly average in $\bar{t}$ of week 10 (solid line) and week 12 (broken line). All age cohorts have an absolute increase in $\bar{t}$, but its smallest for the youngest age cohort. The ratio changes only little for the oldest cohort.

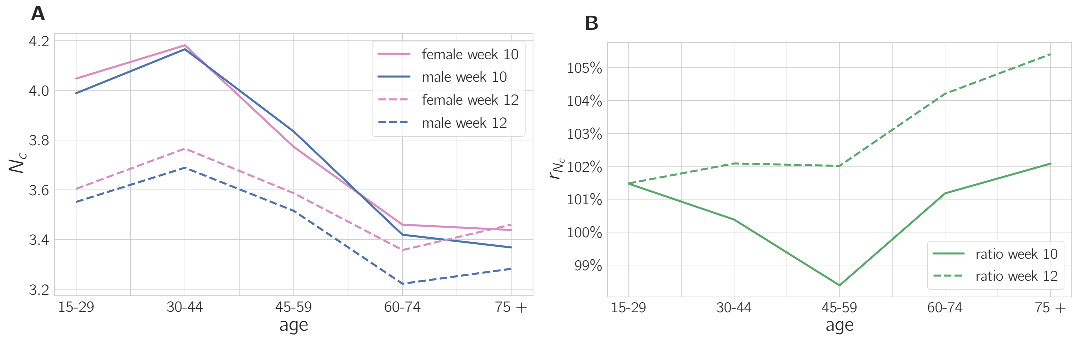

Figure 17. Age profile number of calls N_c. (**A**) Weekly average in N_c of week 10 (solid line) and week 12 (broken line). (**B**) Gender ratio of the weekly average in N_c of week 10 (solid line) and week 12 (broken line). All age cohorts have an absolute increase in N_c, but its smallest for the oldest age cohort. However, the ratio changes only little for the youngest and much for the oldest cohort.

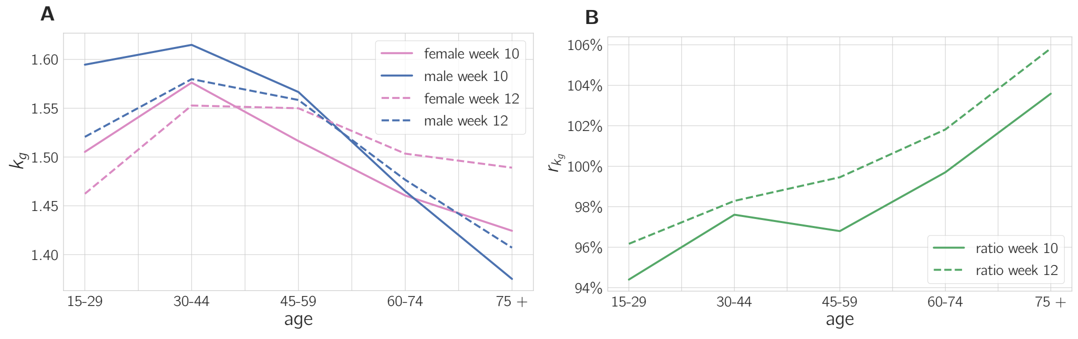

Figure 18. Age profile degree k_i. (**A**) Weekly average in k_i of week 10 (solid line) and week 12 (broken line). (**B**) Gender ratio of the weekly average in k_i of week 10 (solid line) and week 12 (broken line). This curve is interestingly different from the number of calls in Fig. 17. The absolute values of unique communication partners only increase for younger cohorts and decrease for senior cohorts. Nevertheless, gender ratio rises for all age cohorts.

SI Text S6: Statistical significance of gender-ratio-changes across phases

We perform significance tests to ensure that the changes gender ratio in the observed quantities are indeed significant. Gender ratios of different samples of observables (e.g. stationary values during different phases) are compared with a two-sided Mann-Whitney-U test to reject the null hypothesis that they are from the same distribution. The results are shown in Tab. 5 for the overall quantities and in Tab. 6 we report age stratified tests.

For R_G we can show that the reduction from phase I to phases II, III, IV, V and VI is highly significant on weekdays, while the change on weekends is less significant, except for phase IV. The change from before to after Easter, phase III vs. IV is significant as well on weekends and weekends.

Table 5. A Mann Whitney U test was applied to test for significance. We compare the ratio female/male for various metrics of one period against another period, to reject the hypothesis that they are drawn from the same distribution. The following rules were used when assigning the significance stars: $* < 0.05, ** < 0.01, *** < 0.001$

metric	compared periods	Mann-Whitney Weekday	Weekend
R_G [m]	I vs. III	3.94e-10, ***	2.69e-01, -
R_G [m]	I vs. IV	6.19e-08, ***	6.29e-03, **
R_G [m]	I vs. V	3.07e-05, ***	3.95e-01, -
R_G [m]	I vs. VI	5.02e-03, **	2.08e-02, *
R_G [m]	III vs. IV	1.45e-05, ***	2.02e-02, *
S	I vs. III	2.88e-03, **	7.94e-05, ***
S	I vs. IV	6.19e-08, ***	4.37e-01, -
S	I vs. V	3.31e-04, ***	3.16e-02, *
S	I vs. VI	4.19e-04, ***	6.51e-04, ***
S	III vs. IV	9.15e-07, ***	2.02e-02, *
S_i	I vs. III	3.94e-10, ***	2.17e-02, *
S_i	I vs. IV	6.19e-08, ***	6.29e-03, **
S_i	I vs. V	7.49e-07, ***	4.64e-03, **
S_i	I vs. VI	3.27e-01, -	5.78e-04, ***
S_i	III vs. IV	1.31e-01, -	8.96e-02, -
total call duration (MO) [s]	I vs. III	3.93e-10, ***	2.35e-05, ***
total call duration (MO) [s]	I vs. IV	2.63e-07, ***	7.24e-03, **
total call duration (MO) [s]	I vs. V	3.77e-01, -	3.55e-01, -
total call duration (MO) [s]	I vs. VI	2.13e-11, ***	5.12e-02, -
total call duration (MO) [s]	III vs. IV	9.15e-07, ***	9.81e-03, **
total call duration (MT) [s]	I vs. III	3.94e-10, ***	2.35e-05, ***
total call duration (MT) [s]	I vs. IV	6.19e-08, ***	1.91e-02, *
total call duration (MT) [s]	I vs. V	1.28e-04, ***	3.16e-01, -
total call duration (MT) [s]	I vs. VI	3.55e-07, ***	2.35e-02, *
total call duration (MT) [s]	III vs. IV	1.10e-06, ***	4.44e-03, **
degree	I vs. III	5.97e-04, ***	2.36e-05, ***
degree	I vs. IV	5.88e-06, ***	3.55e-01, -
degree	I vs. V	5.21e-04, ***	3.55e-01, -
degree	I vs. VI	1.34e-07, ***	2.22e-03, **
degree	III vs. IV	3.79e-06, ***	2.91e-03, **

Additionally we compare the R_G between phase V and phase VI to check for significant changes after school openings. We perform a two-sided Mann-Whitney-U test to reject the null hypothesis that there is no change. We find a significance level of $p = 4.7 \cdot 10^{-5}$.

Table 6. Significance testing results per gendered age group for selected metrics. We apply a Mann Whitney U test, comparing the ratio female/male for various metrics of one period against another period, to reject the hypothesis that they are drawn from the same distribution. The following rules were used when assigning the significance stars: $* < 0.05, ** < 0.01, *** < 0.001$

metric	compared periods	age group	Mann-Whitney	
		Weekday	Weekend	
R_G [m]	I vs. III	15-29	1.18e-09, ***	1.26e-04, ***
R_G [m]	I vs. III	30-44	1.18e-09, ***	3.42e-04, ***
R_G [m]	I vs. III	45-59	1.68e-09, ***	3.83e-01, -
R_G [m]	I vs. III	60-74	2.29e-02, *	8.68e-04, ***
R_G [m]	I vs. III	75 +	1.18e-09, ***	4.37e-05, ***
R_G [m]	I vs. IV	15-29	1.17e-07, ***	2.19e-03, **
R_G [m]	I vs. IV	30-44	1.17e-07, ***	2.19e-03, **
R_G [m]	I vs. IV	45-59	1.17e-07, ***	1.24e-02, *
R_G [m]	I vs. IV	60-74	1.34e-07, ***	4.28e-01, -
R_G [m]	I vs. IV	75 +	5.54e-02, -	3.01e-02, *
R_G [m]	I vs. V	15-29	3.94e-05, ***	1.82e-01, -
R_G [m]	I vs. V	30-44	3.50e-05, ***	1.51e-01, -
R_G [m]	I vs. V	45-59	7.96e-05, ***	4.76e-01, -
R_G [m]	I vs. V	60-74	3.29e-03, **	4.76e-01, -
R_G [m]	I vs. V	75 +	4.21e-01, -	2.27e-02, *
R_G [m]	I vs. VI	15-29	7.57e-07, ***	2.65e-03, **
R_G [m]	I vs. VI	30-44	1.69e-02, *	6.02e-02, -
R_G [m]	I vs. VI	45-59	1.03e-01, -	1.12e-01, -
R_G [m]	I vs. VI	60-74	1.73e-03, **	3.48e-02, *
R_G [m]	I vs. VI	75 +	1.68e-10, ***	2.11e-01, -
R_G [m]	III vs. IV	15-29	1.93e-01, -	4.72e-01, -
R_G [m]	III vs. IV	30-44	5.56e-04, ***	2.81e-02, *
R_G [m]	III vs. IV	45-59	1.23e-04, ***	9.81e-03, **
R_G [m]	III vs. IV	60-74	6.33e-06, ***	3.85e-02, *
R_G [m]	III vs. IV	75 +	1.58e-06, ***	3.85e-02, *
total call duration (MO) [s]	I vs. III	15-29	7.41e-07, ***	7.49e-05, ***
total call duration (MO) [s]	I vs. III	30-44	1.18e-09, ***	4.37e-05, ***
total call duration (MO) [s]	I vs. III	45-59	1.18e-09, ***	4.37e-05, ***
total call duration (MO) [s]	I vs. III	60-74	1.18e-09, ***	4.37e-05, ***
total call duration (MO) [s]	I vs. III	75 +	1.17e-08, ***	2.44e-01, -
total call duration (MO) [s]	I vs. IV	15-29	2.60e-01, -	2.93e-01, -
total call duration (MO) [s]	I vs. IV	30-44	4.48e-07, ***	6.48e-02, -
total call duration (MO) [s]	I vs. IV	45-59	1.17e-07, ***	4.56e-03, **
total call duration (MO) [s]	I vs. IV	60-74	1.17e-07, ***	2.19e-03, **
total call duration (MO) [s]	I vs. IV	75 +	2.31e-07, ***	2.19e-03, **
total call duration (MO) [s]	I vs. V	15-29	1.02e-02, *	3.93e-02, *
total call duration (MO) [s]	I vs. V	30-44	4.09e-01, -	1.82e-01, -
total call duration (MO) [s]	I vs. V	45-59	1.40e-04, ***	3.36e-01, -
total call duration (MO) [s]	I vs. V	60-74	7.48e-04, ***	2.52e-01, -
total call duration (MO) [s]	I vs. V	75 +	6.78e-04, ***	4.28e-01, -
total call duration (MO) [s]	I vs. VI	15-29	1.03e-07, ***	4.90e-03, **
total call duration (MO) [s]	I vs. VI	30-44	1.02e-12, ***	4.05e-04, ***
total call duration (MO) [s]	I vs. VI	45-59	1.43e-03, **	2.81e-01, -
total call duration (MO) [s]	I vs. VI	60-74	3.78e-01, -	3.75e-01, -
total call duration (MO) [s]	I vs. VI	75 +	3.89e-03, **	2.70e-02, *
total call duration (MO) [s]	III vs. IV	15-29	2.16e-05, ***	2.02e-02, *
total call duration (MO) [s]	III vs. IV	30-44	9.15e-07, ***	4.44e-03, **
total call duration (MO) [s]	III vs. IV	45-59	1.32e-06, ***	6.66e-03, **

Table 6. Continuation:

metric	compared periods	age group	Mann-Whitney	
		Weekday	Weekend	
total call duration (MO) [s]	III vs. IV	60-74	1.57e-06, ***	4.72e-01, -
total call duration (MO) [s]	III vs. IV	75 +	4.49e-01, -	5.19e-02, -

SI Text S7: Additional analyzes on the robustness of the results

We perform additional analyzes with respect to the statistical, geographical and temporal robustness of the results.

Additionally to the median R_G provided in the main text, we provide the 25% (blue) and 75% (green) percentiles in Supplementary Fig. 19, panel A showing the values for women, panel B for men. The quartiles show a similar trajectory to the median, demonstrating a shift of the complete R_G distribution.

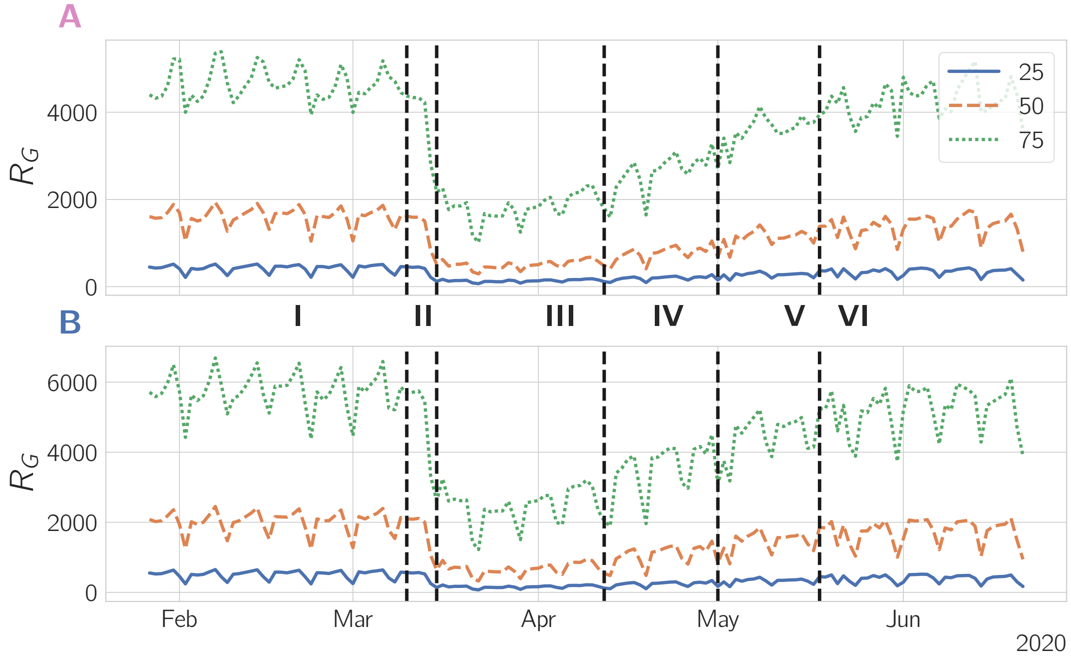

Figure 19. Quartiles for R_G, the blue line shows the 25% , the red the 50% (median) and the green line the 75% percentile. (**A**) Timeseries for women. (**B**) Timeseries for men. The quartiles show similar behavior to the median.

We provide the 25% (blue), median (red) and 75% (green) percentiles of the incoming total call duration in Supplementary Fig. 20, panel A showing the values for women, panel B for men. The quartiles show a similar trajectory to the median, demonstrating a shift of the complete total call distribution.

To understand the robustness of our results across regional differences, for example a potential urban-rural divide, we analyze changes in gender ratios across political districts. We start by analyzing the gender ration of the outgoing call duration. Supplementary Figure 21 shows the average gender ratio of the outgoing call duration for phase I (blue) and III (orange) for every political district. The distribution shifts clearly to higher values in phase III, showing that the effect is present across all districts.

We continue with analyzing R_G. Supplementary Figure 22 shows the histograms for the average R_G gender ratio during period I (blue) and III (orange). The distributions are relatively narrow around the median values given in the main text. The distribution for phase III is shifted only slightly to smaller numbers, showing that the effect is indeed small, as expected from the small change in r_{R_G} shown in Fig. 4 (main text).

Finally, we examine the second lockdown in autumn of 2020. Supplementary Figure 23 shows the gender ratio of R_G from mid January 2020 to mid of May 2021. In reaction to rapidly raising infection numbers, the Austrian government introduced a lock-down "light" on November 3rd, with restaurants, bars and universities closing, but retail and most of Austrian schools staying open. On November 17th the lock-down measures were increased and schools as well as most retail shops were closed[1]. Similar to the first lockdown in phases III to V, we observe a drop in the R_G gender ratio and a subsequent return to

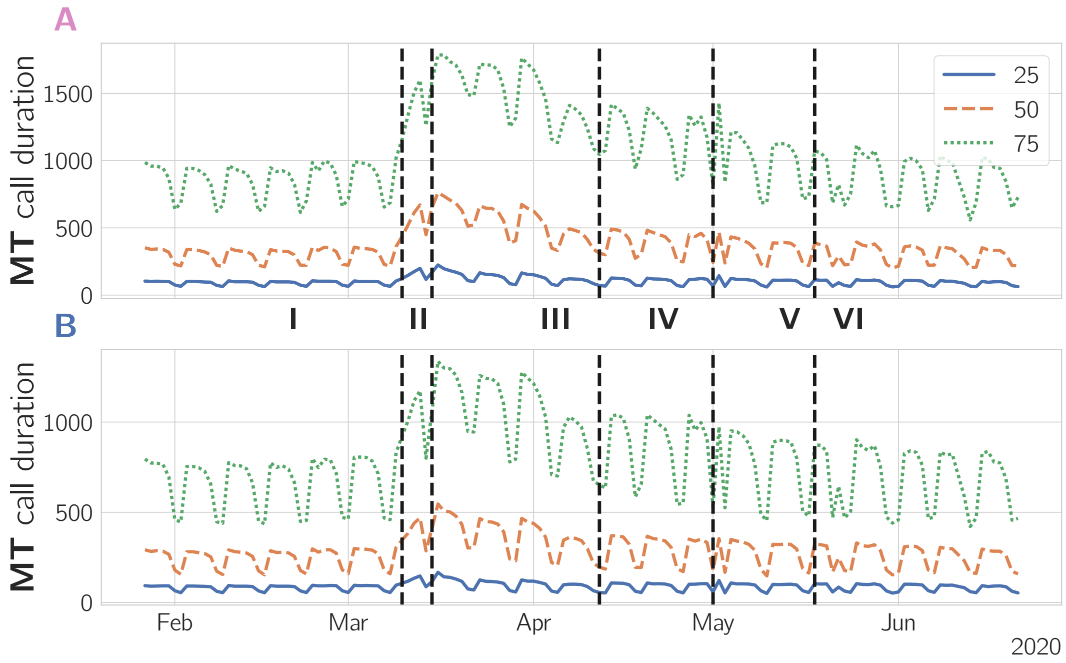

Figure 20. Quartiles for total incoming call duration, the blue line shows the 25% , the red the 50% (median) and the green line the 75% percentile. (**A**) Timeseries for women. (**B**) Timeseries for men. The quartiles show similar behavior to the median.

previous levels. A notable difference for the second lockdown is that the values around the Christmas holidays become more balanced than usual. Without reference data for previous years it is not possible to make conclusions about the interaction of the lock-down measures and the effect of Christmas holidays.

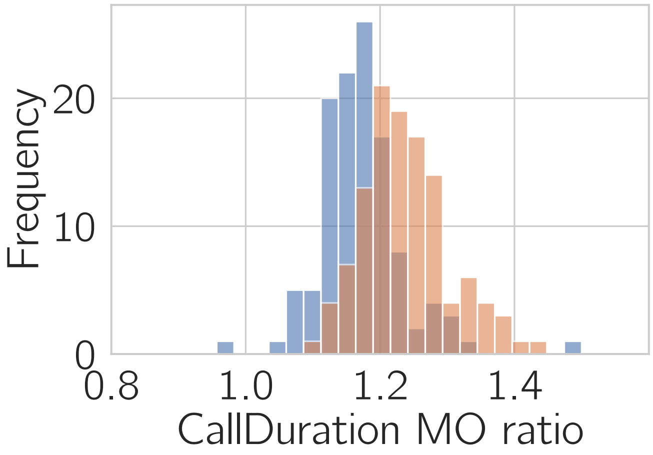

Figure 21. Geographical heterogeneity of the total outgoing call duration gender ratio. Gender ratio of the outgoing call duration for every political district in Austria, phase I in blue and for phase III in orange. The distribution shifts to higher values, showing that the effect described in the main text is consistent across Austrian regions.

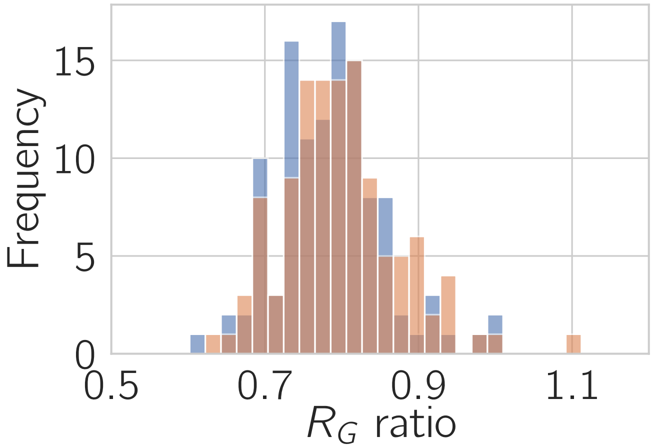

Figure 22. Geographical heterogeneity of the R_G gender ratio. Gender ratio of the radius of gyration for every political district in Austria, phase I in blue and for phase III in orange. The distribution shifts to slightly smaller values.

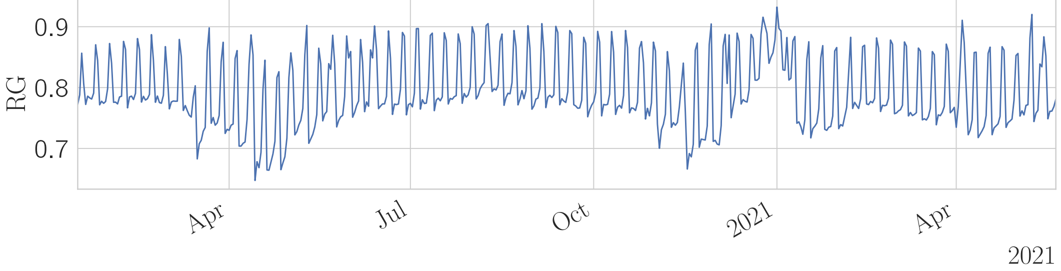

Figure 23. Evolution of the R_G gender ratio one year after the first lock-down. Austria entered a second lock-down in November 2020, again resulting in the gender ratio shifting towards women moving less than men.

SI Text S8: Alternative measure for mobility: entropy

We calculate an alternative measure for mobility, the entropy S_i of the stay time distribution of device i, for a definition see SI Text S3 Methods. A high (low) value of entropy denotes a spread out (concentrated) stay time distribution, i.e. the device was moving a lot (little).

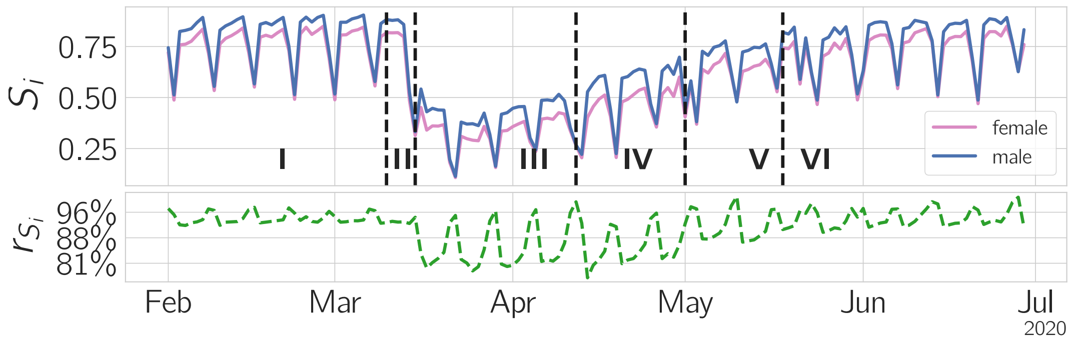

Figure 24. Mobility in Austria, measured by entropy. The upper panel shows entropy S_i for men and women, the lower the gender ratio for entropy r_{S_i}. The drop at the beginning of phase III is very pronounced and so is the change in gender ratio. The weekday gender ratio is shifted towards men moving more and slowly returns to pre-crisis levels.

Figure 24 A shows S_i for both genders across the six phases. We observe a very similar pattern as for the radius of gyration. During the week S_i is high and on weekends it is significantly less. In phase III S_i reaches a minimum and only slowly recovers over the subsequent phases.

The gender ratio of S_i, shown in Fig. 24 B also corroborates the radius of gyration. In phase I the gender ratio is shifted towards men moving more, an effect which is smaller on weekends. During the lock-down in phase III the ratio is shifted strongly towards men and changed little to nothing on weekends. In phase V the ratio recovers to pre-crisis values.

SI Text S9: Long term analysis

A comparison with the data from the same time period of the previous year is available in Fig. 25. The figure shows the ratio of radius of gyration from 2020 and 2019. If R_G was smaller (larger) in 2020 than in 2019, the ratio is less (more) than 100%.

The drastic reduction of movement to approximately 30% compared to the previous year shows the impact of the measures. We also observe that women have a stronger reduction from week 16 to 20.

These results should be interpreted with care, as the accuracy of the localization in the GSM network was improved from 2019 to 2020, potentially resulting in different absolute values of R_G.

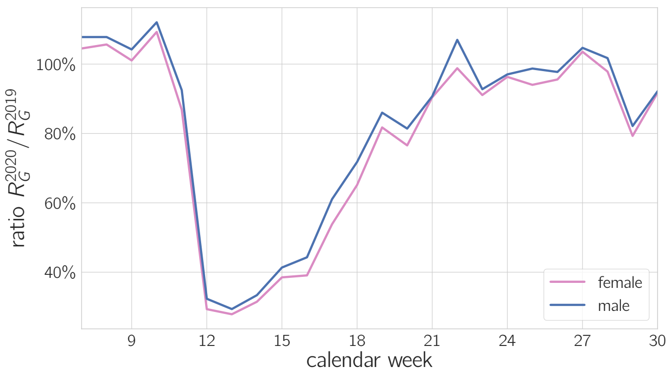

Figure 25. Comparison of the gendered R_G with the same time period of the previous year. We plot the quotient of 2019's R_G with 2020's. During the lock-down the ratio drops below 40%.

SI Text S10: Shopping centers

Here we present the visiting data for a large shopping center in the south of Vienna, as described in the main text. Figure 26 depicts data from the lock-down and is discussed in the main text.

For comparison we analyze the same time from the previous year, 2019, see Fig. 27. In the visitor numbers we find the same weekly pattern as in phase I and a declining trend towards the summer months, which is, nevertheless, much weaker than the reduction in phases III and IV. The gender ratio is quite stable around (or slightly above) 1.

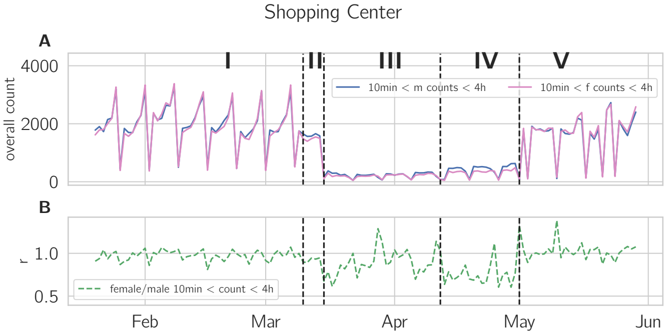

Figure 26. Shopping center in 2020. (**A**) The upper panel shows the visitor count for a large shopping center in the south of Vienna. (**B**) In the lower panel the gender ratio for the visitor count in panel A is shown. The lock-down is prominently visible as a drop in the visitor count and a deviation from equality in the ratio.

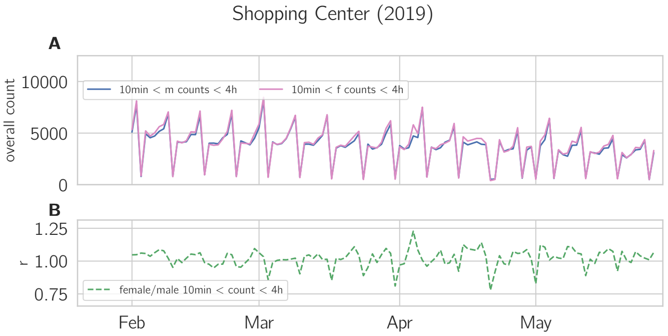

Figure 27. Shopping center in 2019. (**A**) The upper panel shows the visitor count for the same period as in Fig. 26 in 2019 for a large shopping center in the south of Vienna. (**B**) The lower panel displays the gender ratio for the visitor count in panel A. The changes from 2020 are not visible in this figure, however a declining trend from February towards summer is present.

SI Text S11: Statistical significance of gender ratio shopping center and recreational area

We perform statistical tests to check if the observed changes in gender ratio from one phase to another are significant. A two sided Mann-Whitney U test is performed to reject the hypothesis that the values in both phases are drawn from the same distribution.

The changes in gender ratio from phase I to phases III, IV and V are are highly significant on weekdays and not significant on weekends.

For the recreational area we find that changes from phase I to phases III, IV and V are highly significant on weekdays and for phases III and IV significant on weekends with a $p<0.01$ and $p<0.05$ significance level. For the second presented recreational area only the weekday change from phase I to III is significant.

Table 7. A Mann Whitney U test is applied to test for significance. We compare the ratio of female over male count in an area of one phase against another. The following rules were used when assigning the significance stars: *<0.05, **<0.01, ***<0.001.

Point of Interest	compared time periods	Weekday	Weekend
Recreational Area 1	I vs. III	9.60e-08 ***	7.66e-03 **
	I vs. IV	6.85e-05 ***	1.92e-02 *
	I vs. V	6.63e-08 ***	2.81e-01
	III vs. IV	8.90e-02	3.18e-01
Recreational Area 2	I vs. III	1.68e-03 **	4.85e-01
	I vs. IV	7.24e-02	4.79e-01
	I vs. V	2.03e-01	4.86e-01
	III vs. IV	1.59e-01	2.54e-01
Shopping Center	I vs. III	4.04e-07 ***	1.40e-01
	I vs. IV	2.75e-08 ***	1.11e-01
	I vs. V	3.23e-04 ***	4.59e-01
	III vs. IV	4.72e-04 ***	3.63e-02 *

SI Text S12: Recreational areas

Figure 28 shows the recreational area described in the main text. For comparison we also show the relevant time period in the previous year, 2019, in Fig. 29. The limited comparability due to improvements in the network localization can be seen in the lower number of visitors, compared to 2020. However, the increasing trend towards summer is clearly present. The gender ratio is different to 2020, as it is more shifted towards men, however there is no stark change around march 15th, supporting the claim that the change from phase I to III is unique to 2020.

To corroborate these findings we analyze a second recreational area on the other side of Vienna, presented in Fig. 30. There the gender ratio in phase I is typically shifted towards more men present. In the beginning of the lock-down, the gender ratio shifts towards equality, while at the same time more devices are present. A comparison with the previous year, 2019, in Fig. 31 shows that these changes are specific to 2020.

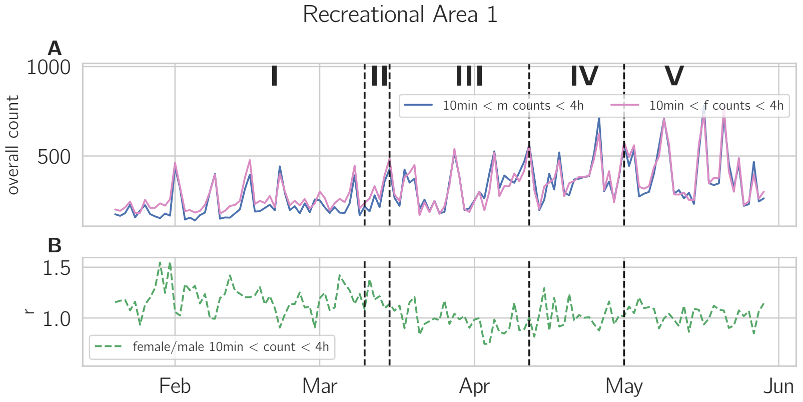

Figure 28. Visitors in a leisure area outside of Vienna, *Kahlenberg*, during the Covid-19 crisis. (**A**) The upper panel shows the counts of men and women present in the defined area. (**B**) The lower panel shows the gender ratio of the counts. The overall counts are unaffected from the lock-down, but the gender ratio changes from being from female-biased to equality.

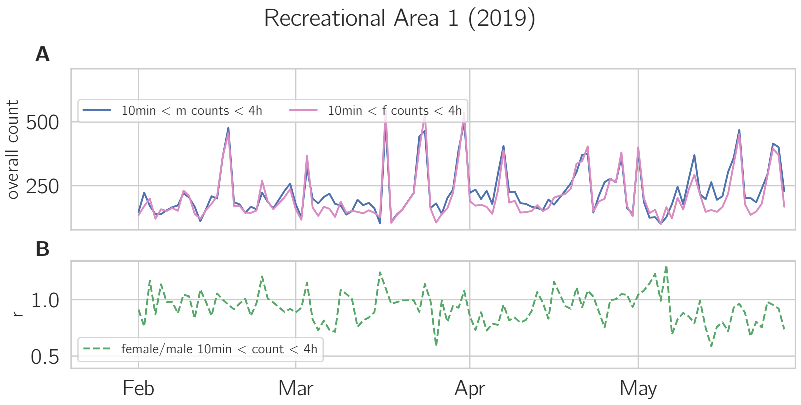

Figure 29. Visitors in the same leisure area as in Fig. 28, but for the same time period in 2019. (**A**) The upper panel shows the number of men and women present, (**B**) the lower panel the gender ratio women over men r. We find a weakly increasing trend towards summer, but the gender ratio stays constant throughout the study period. Note that in contrast to 2020 the gender ratio balanced in February and March.

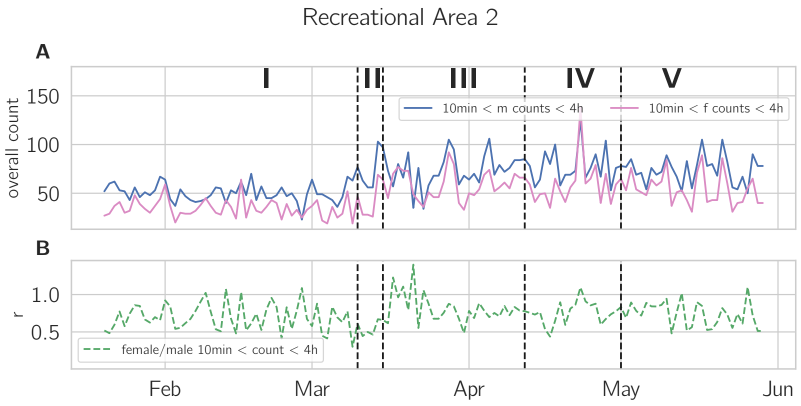

Figure 30. Visitors in another leisure area outside of Vienna, *Lobau*, during the Covid-19 crisis. (**A**) The upper panel shows the counts of men and women present in the defined area. (**B**) The lower panel shows the gender ratio of the counts. The overall counts increase during phase II and stays high until the end of the study period. The gender ratio changes from being from male-biased to being more balanced in phase III.

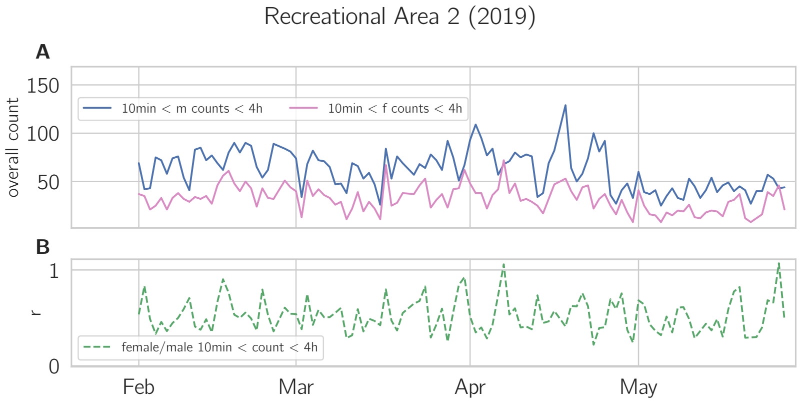

Figure 31. Visitiors in the same leisure area as in Fig. 30, but for the same time period in 2019. (**A**) The upper panel shows the number of men and women present, (**B**) the lower panel the gender ratio women over men r. We do not find an increase in the middle of March, and also gender ratio stays the same throughout the study period.

SI Text S13: Circadian rhythm as observed by other quantities

We investigate the number of calls, down- and uploaded gigabytes per hour as network traffic measures to corroborate the results presented for the call duration in the main text. All investigated quantities show similar patterns, the maximum of the daily activity shifts to the morning, the FWQM gets shorter and the days get more synchronized.

We present the results for the call count per hour in Fig. 32. Panel E epicts how the length of day as measured by FWQM reduces by one hour from 14h before to 13h during the lock-down. Again, the gender ratio of FWMQ does not show any significant differences. The difference in daily circadian rhythm, Δ_{FM}, reduces by about one quarter from phase I to phase III and smoothly recovers until phase IV, see panel F.

Figure 33 shows our results on the downloaded gigabytes per hour. Panels A and B depict how the activity peak in the evening disappears during the lock-down and is only weakly pronounced in the mornings for men. For women the download traffic in panel B does not show a pronounced maximum during the lock-down. The downloaded gigabyte per hour in 33 E show a reduction in FWQM by 40min from 14h 45min to 14h 5min. We only find a slight decrease in Δ_{FM} in the first week of phase III, otherwise the differences in the daily activity patterns of men and women stay constant.

Finally we consider the uploaded gigabytes per hour in Fig. 34. The profile of the network traffic in panels A and B shift from a clear maximum in the evening to a stronger increase in the morning which results in a relatively flat profile during the day in panel B. As shown in panel E the FWQM drops by 45min from 14h 33min to 13h 48min. Panel F shows that Δ_{FM} drops from by approximately one third with the beginning of the lock-down and returns to pre-crisis levels around the beginning of May.

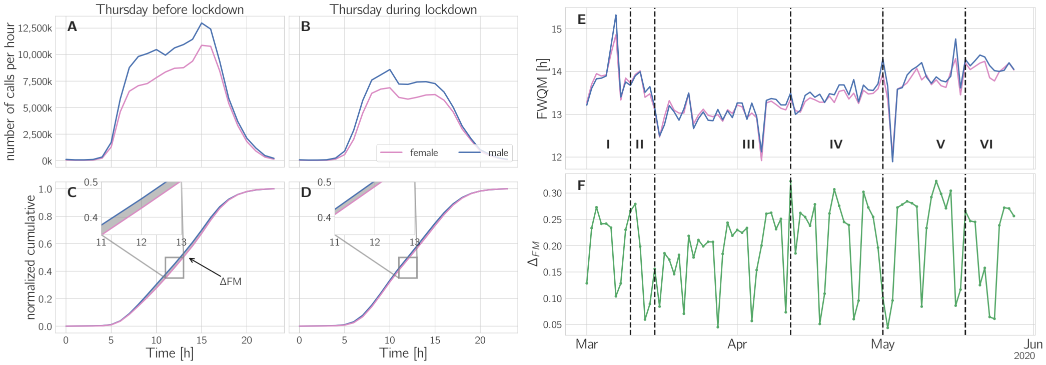

Figure 32. Changes in circadian rhythms during the lock-down measured by number of calls per hour. (**A**) Network traffic on the last Thursday in phase I, March 4th. The horizontal arrow marks the full-width-quarter-maximum length (FWQM). (**B**) Calls per hour on Thursday, March 18th, the first Thursday in phase III. (**C**) Normalized cumulative activity for the day shown in panel A. The inset highlights the difference of the male and female curve. The grey shaded area marks the difference between the circadian rhythm of men and women, denoted Δ_{FM}. (**D**) Same as in C, but for the curve in panel B. (**E**) FWQM for men and women over time. (**F**) The gender ratio r_{FWQM} does not deviate strongly from equality, hence, we show Δ_{FM} over time. For both genders the activity maximum shifts from late afternoon to morning and the length of the activity period is approximately 60 min shorter during the lock-down. The reduction in Δ_{FM} indicates that the circadian rhythms of men and women become more synchronized.

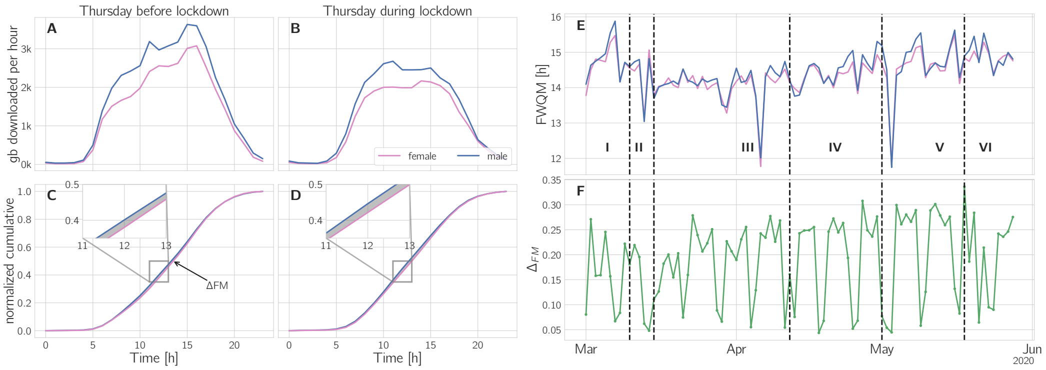

Figure 33. Changes in circadian rhythms during the lock-down measured by downloaded gigabytes per hour. (**A**) Network traffic on the last Thursday in phase I, March 4th. The horizontal arrow marks the full-width-quarter-maximum length (FWQM). (**B**) Downloaded gigabytes per hour on Thursday, March 18th, the first Thursday in phase III. (**C**) Normalized cumulative activity for the day shown in panel A. The inset highlights the difference of the male and female curve. The grey shaded area marks the difference between the circadian rhythm of men and women, denoted Δ_{FM}. (**D**) Same as in C, but for the curve in panel B. (**E**) FWQM for men and women over time. (**F**) As r_{FWQM} does not deviate strongly from equality, we display Δ_{FM} over time. For both genders the activity maximum in the late afternoon disappears and the length of the activity period is approximately 40 min shorter during the lock-down. A slight reduction in Δ_{FM} indicates that the circadian rhythms of men and women become more synchronized.

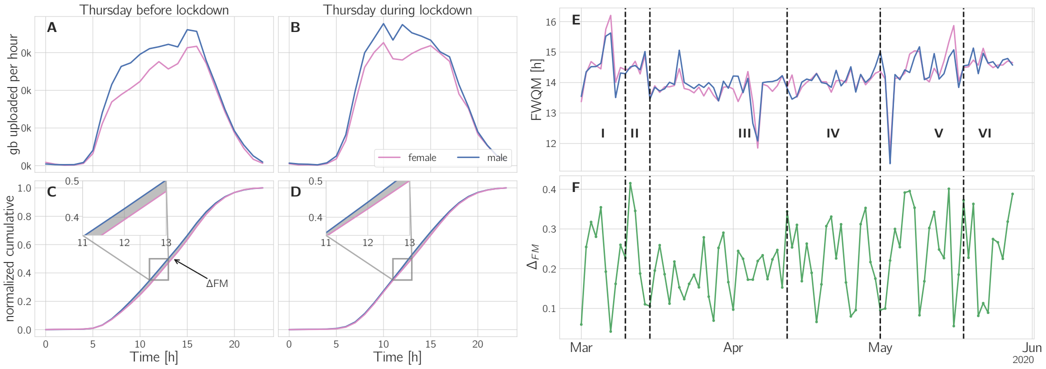

Figure 34. Changes in circadian rhythms during the lock-down measured by uploaded gigabyte per hour. (**A**) Network traffic on the last Thursday in phase I, March 4th. The horizontal arrow marks the full-width-quarter-maximum length (FWQM). (**B**) Uploaded gigabytes per hour on Thursday, March 18th, the first Thursday in phase III. (**C**) Normalized cumulative activity for the day shown in panel A. The inset highlights the difference of the male and female curve. The grey shaded area marks the difference between the circadian rhythm of men and women, denoted Δ_{FM}. (**D**) Same as in C, but for the curve in panel B. (**E**) FWQM for men and women over time. (**F**) As r_{FWQM} does not change much from one, we show Δ_{FM} over time. For both genders the activity maximum shifts from late afternoon to morning and the length of the activity period is approximately 45 min shorter during the lock-down. The reduction in Δ_{FM} indicates that the circadian rhythms of men and women become more synchronized.

References

1. Desvars-Larrive, A. *et al.* A structured open dataset of government interventions in response to COVID-19. *Sci. Data* **7**, 1–9, DOI: 10.1038/s41597-020-00609-9 (2020).

2. Statistik Austria. Bevölkerung nach alter und geschlecht. http://www.statistik.at/web_de/statistiken/menschen_und_gesellschaft/bevoelkerun (2021). Accessed 7 June 2021.
